# Biomarkers of Exposure to Secondhand and Thirdhand Tobacco Smoke: Recent Advances and Future Perspectives

**DOI:** 10.3390/ijerph15122693

**Published:** 2018-11-29

**Authors:** Sònia Torres, Carla Merino, Beatrix Paton, Xavier Correig, Noelia Ramírez

**Affiliations:** 1 Department of Electronic Engineering, Universitat Rovira i Virgili, Països Catalans 26, 43007 Tarragona, Spain; sonia.torres@urv.cat (S.T.); carlamelissa.merino@urv.cat (C.M.); beatrix.paton@urv.cat (B.P.); xavier.correig@urv.cat (X.C.); 2 Institut d’Investigació Sanitària Pere Virgili, Escorxador s/n, 43003 Tarragona, Spain; 3 CIBERDEM, Spanish Biomedical Research Centre in Diabetes and Associated Metabolic Disorders, Carlos III Health Institute, Monforte de Lemos 3-5, 28029 Madrid, Spain

**Keywords:** environmental tobacco smoke, secondhand smoke, thirdhand smoke, tobacco exposure biomarkers, biomonitoring

## Abstract

Smoking is the leading preventable disease worldwide and passive smoking is estimated to be the cause of about 1.0% of worldwide mortality. The determination of tobacco smoke biomarkers in human biological matrices is key to assess the health effects related to the exposure to environmental tobacco smoke. The biomonitoring of cotinine, the main nicotine metabolite, in human biofluids—including urine, serum or saliva—has been extensively used to assess this exposure. However, the simultaneous determination of cotinine together with other tobacco biomarkers and the selection of alternative biological matrices, such as hair, skin or exhaled breath, would enable a better characterization of the kind and extent of tobacco exposure. This review aims to perform a critical analysis of the up-to-date literature focused on the simultaneous determination of multiple tobacco smoke biomarkers studied in different biological matrices, due to the exposure to secondhand smoke (SHS) and thirdhand smoke (THS). Target biomarkers included both tobacco-specific biomarkers—nicotine and tobacco specific nitrosamine biomarkers—and tobacco-related biomarkers, such as those from polycyclic aromatic hydrocarbons, volatile organic compounds, metals and carbon monoxide. To conclude, we discuss the suitability of determining multiple biomarkers through several relevant examples of SHS and THS exposure.

## 1. Introduction

Passive smoking is estimated to be the cause of about 1.0% of worldwide mortality, responsible for approximately 603,000 deaths each year among children and adults, a number which has been increasing over the years [1]. Environmental tobacco smoke (ETS), most commonly called secondhand smoke (SHS), is a complex and reactive mixture made up of the mainstream smoke exhaled by the smokers and sidestream smoke emitted from the burning tobacco diluted with ambient air. This mixture contains over 4700 chemicals including hazardous amines, carbonyls, hydrocarbons or metals among others [2,3,4]. SHS exposure can cause several illnesses in nonsmokers including ischaemic heart diseases in adults and lower respiratory infections and asthma in adults and children, among other adverse health effects [1]. Moreover, the International Agency for Research on Cancer (IARC) classifies 63 chemicals reported in mainstream tobacco smoke as carcinogenic, 11 of them are known as human carcinogens with a proven role on the development of different types of cancer including lung and bladder cancer [2].

Nevertheless, SHS is not the only source of exposure for nonsmokers to tobacco smoke components. Most of the smoke gases and particles of SHS deposit, age and remain for long periods of time in fabrics, surfaces and dust forming the so-called thirdhand smoke (THS), a less studied source of exposure to tobacco smoke toxicants [5,6]. THS components not only remain on surfaces and in settled dust, they can also be re-emitted into the gas phase or even react with oxidants and other atmospheric compounds to yield secondary contaminants, some of them with increased toxicity. This is the case of nicotine, which deposits almost entirely on indoor surfaces, where it can react with ozone, nitrous acid and other atmospheric oxidants producing carcinogenic tobacco-specific nitrosamines (TSNAs) [7]. To date, dozens of toxicants have been identified in THS including tobacco specific toxicants—such as nicotine, and TSNAs—as well as tobacco related toxicants including volatile N-nitrosamines, aromatic amides, polycyclic aromatic hydrocarbons (PAHs) and volatile carbonyls [8,9,10,11,12]. Exposure to THS causes numerous alterations in organ and cellular systems of mouse models, including lung and liver damage, several metabolic effects and signs of hyperactivity [13,14,15]. THS extracts also inhibit cell proliferation and cause DNA strand breaks and oxidative damage in DNA and mitochondria [16]. Pathways of exposure to THS are mainly non-dietary ingestion and dermal absorption, although inhalation of resuspended particles may also occur. Consequently, THS could be one of the major pathways of children exposure to tobacco smoke toxicants. Despite these emerging evidences on THS toxicity and carcinogenicity, this way of exposure to tobacco smoke contaminants is still unrecognized by most of the population and it has been omitted in public health and environmental policies.

The determination of biomarkers of tobacco chemicals is key to assess the health effects related to SHS and THS exposure. The biomonitoring of cotinine, the main nicotine biomarker, in urine, blood and saliva has been the preferred option to assess the kind, extent and frequency of tobacco smoke exposure. However, SHS and THS exposure results in the uptake of complex mixtures of toxicants, therefore, a wide range of tobacco specific and related biomarkers could be assessed. Tobacco exposure biomarker concentrations can vary depending on which source of exposure, SHS or THS, is predominant. Many other factors can induce variation, such as the life-stage of the target population or their race, among others that will be discussed below. Therefore, the simultaneous study of multiple tobacco smoke biomarkers and the analysis of different biological matrices would provide a wider assessment of the extent of tobacco smoke exposure that may help to better understand its implications in human health.

Hence, this review aims to provide a critical overview on the assessment of exposure to tobacco smoke in nonsmokers, including both SHS and THS exposures, through the determination of cotinine, as the most renowned tobacco-specific biomarker, together with other specific and related tobacco biomarkers. In recent years, an acceptable number of studies have focused on this multiple approach to assess both SHS and THS exposure. In 2013, a former publication reviewed the use of tobacco-specific biomarkers to study SHS exposure [17]. In the present paper we aimed to establish for the first time a joint discussion on the convenience of this multiple biomarkers approach to assess both, SHS and THS exposures, through the review of the most up-to-date bibliography in this respect. To that end, this report includes those studies published from 2012 to March 2018 that focus on tobacco smoke biomarkers from conventional tobacco smoke and waterpipe smoke. Here, different aspects are covered, from the advantages and disadvantages of the different biological matrices, to a general introduction about the biomarkers studied in this period of time, metabolism, general toxicity of their precursors and main ranges of concentrations. To conclude, we discuss the suitability of the determination of multiple biomarkers for assessing the kind and extent of SHS and THS exposure through several relevant examples of applications.

## 2. Selection of Papers

For the purpose of this review, we have selected original research publications published from 2012 to March 2018 with content based on the exposure to SHS and THS from tobacco combustion, in order to cover the recent trends in this topic. Reviews and articles exclusively based on the analysis of previous survey data were excluded and also those from e-cigarettes. Various searches were combined to identify relevant literature in the Web of Science (using the Web of Science^®^ Core Collection WoS, Thomson Reuters; http://webofscience.com) using the keyword “cotinine” AND multiple combinations of the following keywords: “environmental”, “secondhand” OR “second-hand”, “thirdhand” OR “third-hand”, “tobacco”, “smoke” and “cigarette”. The papers obtained in this search were then individually revised to meet the inclusion criteria. To be included in this review, articles had to: (A) Provide original data from observational or experimental studies in human nonsmokers exposed to SHS and/or THS; (B) provide levels of other tobacco smoke biomarkers besides cotinine, including either specific and non-specific biomarkers of SHS and THS exposure. A further revision of the preselected papers was performed to assess the quality of the studies, excluding those based on isolated observations, and those not addressing quality parameters of the reported concentrations.

Table S1 in the Supplementary Information summarizes the 44 papers included in this review, as well as their most relevant information, including the number and main characteristics of the target population, the biological matrices analyzed, the determined biomarkers and the ranges of the reported concentrations in nonsmokers exposed to SHS and/or THS. To complete the discussion, biomarker concentrations of smokers have been included, when available.

## 3. Biological Matrices

Biological matrix selection is one of the key points for a rigorous characterization of the kind and extent of the exposure. The selected matrix or matrices will depend on the aim and nature of the study, the life stage of the target population, the type of exposure and also the availability of robust analytical methods that allow a reliable determination of the biomarkers of interest in a concrete matrix. Commonly, the reasonable choice is to analyze the least-invasive matrix in which the target biomarkers are more easily determined with the available analytical methods and to choose a matrix that will provide a broader assessment of the exposure. In the papers reviewed here, urine, saliva and blood have been the preferred matrices for SHS and THS human biomonitoring, but there is an increasing interest in alternative matrices, such as hair, skin and, to a lesser extent, exhaled breath.

Urine has been the most widely used biological matrix to asses tobacco smoke exposure, since it is a non-invasive biofluid which can be easily obtained. Besides, it accumulates higher concentrations of some biomarkers in comparison with other biofluids, making urine the most sensitive matrix for the assessment of both SHS and THS exposure. The main disadvantages are that renal diseases or the use of certain prescription drugs may interfere with the clearance of urinary biomarkers and that urine dilution adjustments, such as creatinine or specific gravity adjustments, are needed prior to biomarker concentration comparison across samples [17]. Most of the urinary biomarkers are also excreted as glucuronide conjugates. The deglucuronization to the original form prior to the analysis would depend on the application and aim of the study. As an example, the concentration of free urinary cotinine correlates better with serum cotinine than with total cotinine concentration (including both free cotinine and cotinine glucuronide measured after deglucuronization) [18], whilst the evaluation of total 4-(methylnitrosamino)-1-(3-pyridyl)-1-butanol (NNAL) concentration in low SHS and THS exposures is preferred because NNAL glucuronide is excreted in higher concentrations (by a factor of about two, ethnicity dependent) than urinary free NNAL [19].

Blood does not require dilution adjustments, but its collection is more invasive and tobacco biomarkers are less concentrated than in urine (i.e., cotinine concentrations in serum are about four-fold to six-fold lower than in urine) making blood less suitable for the assessment of THS exposure and low and intermittent SHS exposure [17]. Blood biomonitoring can be performed in different formats: Whereas plasma and serum are the most commonly used formats, whole blood is appropriate for the evaluation of metals because they are distributed between non-cellular and intra-cellular compartments [20]. Furthermore, dried blood spots (DBS) and cord blood are becoming popular for the screening of early-life exposure to tobacco smoke toxicants [21,22].

Saliva is a valuable alternative matrix to determine SHS and THS biomarkers, as it is non-invasive and easy to obtain. For smokers and nonsmokers recently exposed, salivary cotinine values correlate well with blood cotinine and, therefore, saliva collection is a feasible alternative when collecting blood samples is not a viable option, or when multiple measurements are required in a limited period of time [17].

Hair is the most likely used matrix to determine long-term SHS and THS exposure. Compared to other biological matrices, it is less affected by daily exposure and metabolism variability than other biological matrices allowing a more robust comparison [23]. The main advantages of hair are that it is a non-invasive matrix, easy to collect and can be stored at room temperature up to five years [24]. There seems to be a significant role played by hair melanin with basic and less polar compounds being selectively enriched, which embeds them in hair as it grows [25].

Although the major intake of tobacco smoke toxicants is through the inhalation of SHS, the biomonitoring of toxicants accumulated in the skin is especially relevant in the case of THS exposure where nonsmokers are exposed to smoke toxicants bound to fabrics, clothing, settle dust and surfaces. Even though the skin performs an effective barrier function [26], nicotine can be dermally absorbed and transported to the dermal blood supply [27].

Finally, exhaled breath condensate is a biological matrix of increased interest in epidemiology because it provides an immediate, non-invasive method of assessing smoking status. In the context of tobacco smoke exposure, the determination of CO in exhaled breath could be used as a short-term SHS biomarker, though, other sources of pollution including exhaust gases may cause elevations in the fractional concentrations of CO in expired air [28].

**Figure 1:**
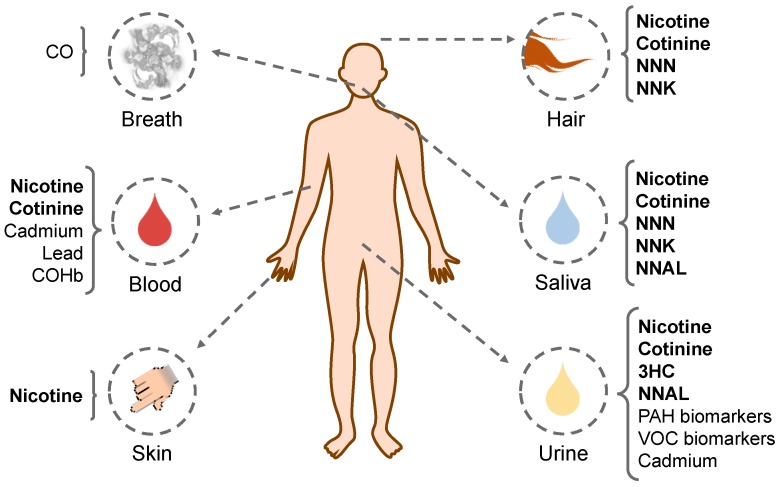
Summary of the biological matrices studied in this review and the tobacco smoke biomarkers determined in each matrix. Tobacco specific biomarkers are indicated in bold. CO: carbon monoxide; NNN: N′-nitrosonornicotine; NNK: 4-(methylnitrosoamino)-1-(3-pyridyl)-1-butanone; COHb: Carboxyhemoglobin; 3HC: *trans*-3’-hydroxycotinine; NNAL: 4-(methylnitrosamino)-1-(3-pyridyl)-1-butanol; PAH: polycyclic aromatic hydrocarbon; VOC: volatile organic compounds.

In Figure 1, the summary of the reviewed biological matrices analyzed in the papers and the tobacco smoke biomarkers determined in each matrix are shown.

## 4. Tobacco-Specific Biomarkers

Tobacco-specific biomarkers are those derived from chemicals exclusively from tobacco smoke: Nicotine and tobacco specific nitrosamines. This section describes the specific tobacco smoke biomarkers that have been determined in the research papers reviewed here, their mechanism of formation, as well as the toxicity of their precursors, with the aim of highlighting their toxicological relevance. Table 1 shows the biomarkers reviewed here, their half-life times and also the main toxicological information of the biomarker precursors, including the either carcinogenic and non-carcinogenic data. Table 2 summarizes the most common ranges of concentrations of the studied biomarkers in each biological matrix in accordance with the reviewed references. These concentrations have been classified regarding the type of tobacco smoke exposure: Smokers, SHS exposure and THS exposure. The column “No exposure”, includes the biomarkers found in a non-exposed population. This table only includes those biomarkers with information in more than one type of tobacco exposure.

### 4.1. Nicotine

Nicotine is the main alkaloid found in tobacco leaves and an exclusive marker of tobacco smoke exposure. During smoking, nicotine is emitted in both gas and particulate phases and rapidly absorbed into the bloodstream. It is then distributed along body tissues and organs, such as the liver which metabolizes nicotine into other compounds. Although nicotine is not considered to be a carcinogen by the International Agency for Research on Cancer (IARC), it can participate in carcinogenesis through inhibition of apoptosis and cell proliferation [71]. Moreover, nicotine is involved in tobacco addiction, promotion of inflammation, adverse effects in the vascular system, reproductive toxicity and alterations in fetus brain development [24,72].

Few studies have focused on the determination of nicotine in human biofluids, mainly because of the low nicotine half-life times (t_1/2_) (11 h and 2 h in urine and blood, respectively) [73]. However, nicotine is a useful biomarker for long-term tobacco smoke exposure in hair, where it remains unmetabolized and, consequently, as hair grows over the months, tobacco exposure is “recorded” over long periods of time [74]. As shown in Table 2, nicotine in hair can be found in 10 to 100 times higher concentrations than cotinine typically ranging from 2.01 to 79.3 ng/mg in smokers [35] and from 0.08 to 5.02 ng/mg [23,35,36,37,38,39,40] in a SHS-exposed population. Hair nicotine concentrations also highly correlated with airborne nicotine and cotinine in urine, thus confirming its suitability as an alternative tobacco smoke exposure biomarker. Furthermore, since nicotine in hair is less affected by daily variability, possible cutoff values have been proposed to distinguish active smokers from SHS-exposed nonsmokers, such as 5.68 ng/mg (sensitivity, 94.2%; specificity, 87.0%) [64].

Besides, the determination of nicotine in skin, especially in hands and fingers, could be also an excellent indicator of the kind and extent of tobacco smoke exposure. Skin nicotine concentrations reached values up to 1160 ng/wipe in smokers [41] and up to 48.9, 46.1 and 17.7 ng/wipe in nonsmokers exposed to SHS, THS and non-exposed, respectively [42,43]. Similarly to hair, the accumulation of nicotine in the hands of nonsmokers also correlated with airborne nicotine and urinary cotinine, making skin nicotine a feasible biomarker to monitor low SHS and THS exposure.

### 4.2. Nicotine Metabolites

**Figure 2:**
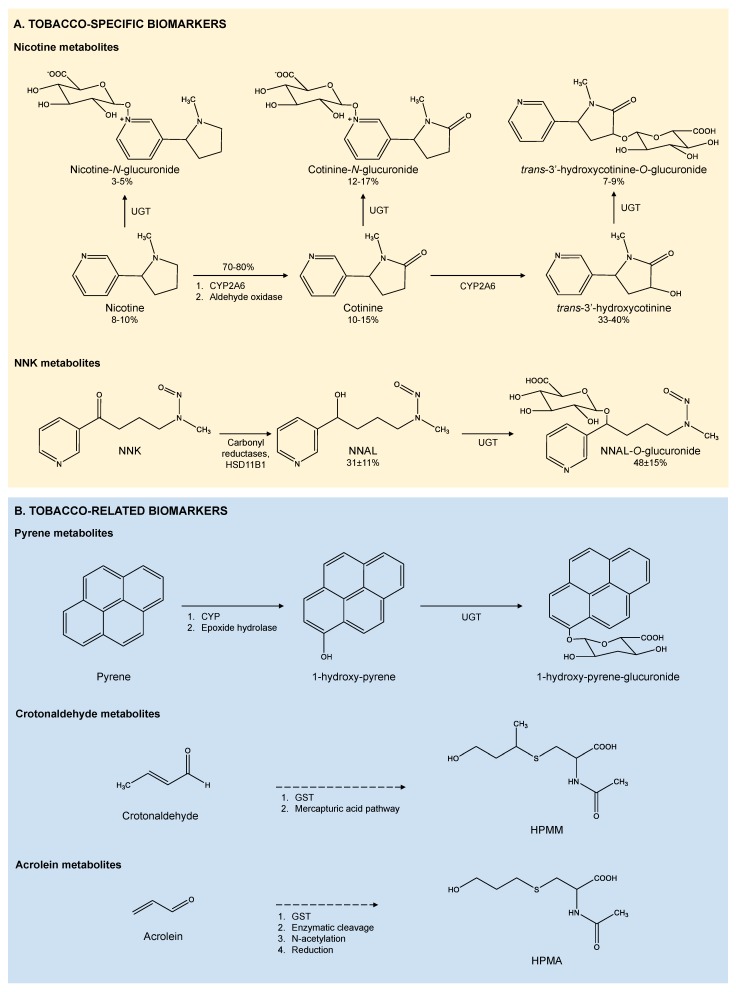
(**A**) Mechanisms of formation of the tobacco smoke specific biomarkers studied in this review, including the main ranges of transformation, expressed in percentage (%) [24,84]. (**B**) Mechanisms of formation of some tobacco smoke-related biomarkers reviewed here [85,86,87].

Nicotine metabolism, summarized in Figure 2A, depends on several factors including ethnicity, gender, age, genetics, pregnancy or several diseases, such as liver or kidney disease [24]. Around 70% to 80% of nicotine is transformed into its main metabolite, cotinine, by two enzymatic reactions [75] carried out by cytochrome P450 2A6 (CYP2A6) in combination with a cytoplasmic aldehyde oxidase [76,77]. The higher persistence of cotinine (t_1/2_ of 15 h in saliva, 16 h in blood and 3–4 days in urine, shown in Table 1), together with the wide range of available analytical methods, makes it the most widely used biomarker to assess tobacco smoke exposure [75,78]. However, it is estimated that only around 10% to 15% of cotinine is found in smokers’ urine because most of cotinine is converted into other metabolites [75], mainly *trans*-3′-hydroxycotinine (3HC), also through a CYP2A6 mediated reaction [79]. The occurrence of 3HC in urine is 33% to 40% and the average half-life of 3HC is similar in both plasma and urine (an average of 6.6 h and 6.4 h, respectively) [80]. Nicotine and its metabolites could also be transformed into N-quaternary glucuronides by the uridine diphosphate-glucuronosyltransferase (UGT). These glucuronides are present in urine with occurrences of 3% to 5%, 12% to 17% and 7% to 9% for nicotine, cotinine and 3HC glucuronides, respectively [73], but may be partially hydrolyzed after sample collection [81]. Hence, the enzymatic hydrolyzation of the glucuronides conjugates is the common procedure prior to the samples analysis.

Cotinine concentrations have been mainly monitored in biofluids. Urine, blood and saliva cotinine concentrations have been used to both establish cutoff values to distinguish active smokers from nonsmokers and characterize the type and extent of the exposure. The typical urinary cotinine cutoff value is 30 ng/mL. Urinary cotinine concentrations usually range from 34.5 to 489 ng/mL for smokers [46,47], from 0.25 to 30 ng/mL for SHS exposed nonsmokers [22,36,42,45,46,48,49,50], up to 5 ng/mL for THS exposed nonsmokers [41,43,44] and around 0.88 ng/mL in non-exposed nonsmokers [43]. Nevertheless, acute exposure to SHS can raise urinary cotinine concentrations to levels similar to those reported in smokers’ concentrations. This is for instance the case of nonsmoker workers of bars and restaurants without smoking bans that presented mean urinary cotinine concentrations in the range of 35.9 to 61.2 ng/mL [44,47].

In serum and plasma, cotinine cutoff values are typically 10 or 15 ng/mL [51,53,54,55,57,58]. Common cotinine levels ranged from 0.015 to 14.6 ng/mL for SHS exposed nonsmokers [49,53,54,55,56,57,58], whereas cotinine levels in smokers can be more than one order of magnitude higher [51,52,53,54,55,57,58]. Non-exposed nonsmokers did not present quantifiable cotinine values. The incidence of several illnesses may affect serum cotinine levels of nonsmokers. For instance, nonsmoker adults with self-reported asthma from the 2007–2008 U.S. National Health and Nutrition Examination Survey (NHANES) presented serum cotinine concentrations reaching up to 57 ng/mL [51].

Salivary cotinine is an alternative to blood worth considering: Concentration in saliva is usually between 15% and 40% higher than in blood because cotinine molecules are small, relatively water soluble and present minimal protein binding in the blood [82]. The interpretation of saliva cotinine can be limited by variability across individuals caused by the effect of age, gender, race, oral pH, type of diet, dehydration or drug treatment [17]. The usual salivary cotinine cutoff value was 13 ng/mL to distinguish between active smokers from nonsmokers, whilst 10 ng/mL was useful to distinguish low and high SHS exposure [39]. More recently, Lam et al. examined the associations between measured SHS exposure and mental health. Salivary cotinine levels were used to categorize the studied population into different groups according to the level of exposure: Low SHS exposure (0.1–0.3 ng/mL), moderate SHS exposure (0.4–0.7 ng/mL) and high SHS (0.8–14.9 ng/mL) [59]. As shown in Table 2, salivary cotinine concentration ranged between 0.04 and 14.9 ng/mL in nonsmokers exposed to SHS [36,38,39,40,59,61,62,63], and reached up to 653 ng/mL in active smokers [38,39,59,60]. Salivary cotinine concentrations increase even after a short time of SHS exposure, thus demonstrating the suitability of salivary cotinine as a short-term SHS exposure biomarker [61].

3HC urinary concentration values are usually three-to four-fold higher than those found for urinary cotinine and, therefore, the determination of urinary 3HC would provide a more sensitive measurement of tobacco smoke exposure [73,83]. Nevertheless, the study of 3HC is mainly used in smoker cohorts and only one of the studies that met our selection criteria have focused on the determination of this biomarker in nonsmokers. Mean 3HC concentrations were ca. 10 fold-higher in smokers than in SHS exposed nonsmokers (654 vs. 60.8 μg per g of creatinine (μg/g cr), respectively) [65].

### 4.3. Tobacco-Specific Nitrosamines (TSNAs)

During tobacco curing and burning, nicotine reacts to form tobacco-specific nitrosamines (TSNAs), a leading class of carcinogens in tobacco products. TSNAs can also be formed by the oxidation of residual nicotine deposited in dust particles and surfaces through the reaction with ozone, nitrous acid and other atmospheric oxidants [7]. Therefore, the determination of TSNAs biomarkers is especially relevant to evaluate THS exposure. As shown in Table 1, N′-nitrosonornicotine (NNN) and 4-(methylnitrosoamino)-1-(3-pyridyl)-1-butanone (NNK) are considered carcinogenic for humans (Group 1) by the IARC [88] with an inhalation unit risk of 4.0 × 10^−4^ (µg/m^3^)^−1^ and 5.2 × 10^−3^ (µg/m^3^)^−1^, respectively. Carcinogenesis of NNN and NNK comes through their metabolic activation mainly conducted by cytochrome P450, generating reactive species that form adducts with DNA [89]. Studies performed in small rodents showed that NNK induced tumors in the lungs, nasal cavities, trachea and liver, while NNN produced tumors in esophagus as well as in lungs, nasal cavities and trachea [90,91]. After absorption, carbonyl reductases and 11β-hydroxysteroid dehydrogenase type 1 (HSD11B1) rapidly convert NNK to its main metabolite 4-(methylnitrosamino)-1-(3-pyridyl)-1-butanol (NNAL), shown in Figure 2A, which is considered to have similar adverse health effects as its precursor [92]. Urinary levels of total NNAL and total NNN have been linked with lung and esophageal cancer risk, respectively [93,94]. NNAL can be transformed to NNAL-glucuronide by UGT enzymes prior to body detoxification [84], mainly into NNAL-*O*-glucuronide in urine [95]. The higher occurrence of NNAL in urine (i.e., urinary NNAL levels are about 30-fold higher than urinary NNN levels) and its higher half-life time (40–45 days against 2.6 h for NNAL and NNK, respectively, shown in Table 1), makes urinary NNAL a good biomarker of long-term and intermittent exposure to tobacco smoke [84,88]. Besides, as SHS ages, nicotine levels rapidly decline but NNK levels increase confirming the suitability of urinary NNAL as a more reliable biomarker of THS exposure than nicotine metabolites. Mean urinary NNAL concentrations were 80.9–405.5 pg/mL in active smokers [50,51,58], 5.9–20.1 pg/mL for high SHS exposure [44,48,58,66], 0.95–2.21 pg/mL for low SHS exposure [36,50,51,54,61], 2.7–6.7 pg/mL for THS exposure [42] and 0.81 pg/mL for no exposure [43]. Recently, Benowitz et al. estimated a urinary NNAL cutoff value to distinguish between active smokers and nonsmokers exposed to SHS. For a cotinine cutoff of 30 mg/L, the estimated NNAL was 14.4 pg/mL (10.2 pg/mg creatinine), with 94.6% sensitivity and 93.4% specificity [50].

Data from the 2011–2012 NHANES showed that urinary NNAL was over 20 times higher for nonsmokers exposed to SHS at home compared to those non-exposed. Moreover, irrespective of smoking status, non-Hispanic Asian American presented lower biomarkers concentrations compared to both non-Hispanic whites and non-Hispanic blacks thus corroborating differences in the elimination kinetics of nicotine/cotinine and NNAL [58]. Data from NHANES 2011–2012 was also used to assess NNK exposure by age group and ethnicity by measuring urinary NNAL in 4831 nonsmokers. Among all non-tobacco users, significantly higher geometric means and 95th percentiles of urinary total NNAL were observed among children aged 6 to 19 years old (2.43 (1.96–3.02) pg/mL) vs. adults aged >20 years (1.38 (1.21–1.57) pg/mL). Among these nonsmokers, non-Hispanic Blacks had higher urinary levels of NNAL (volume-base and creatinine corrected) than other ethnicity groups [54].

Nevertheless, non-metabolized TSNAs could be the preferable choice in other biological matrices. In this sense, a recent study performed by Perez-Ortuño et al. showed that NNK was the most concentrated TSNA in hair of nonsmokers exposed to SHS, with a mean concentration of 2.1 pg/mg, correlating well with nicotine and cotinine. Consequently, NNK could be the most suitable hair biomarker of cumulative exposure to TSNAs [23]. Conversely, the same group of researchers found that NNN was the most prevalent TSNAs in nonsmokers’ saliva samples, with a mean concentration of 5.3 pg/mL. The salivary NNN/cotinine ratio confirmed the relative NNN increase in SHS exposure. Considering that NNN is associated with esophageal and oral cavity cancers, the authors proposed the monitoring of salivary NNN to assess the cancer risk associated with exposure to tobacco smoke.

## 5. Tobacco-Related Biomarkers

SHS and THS exposure results in the uptake of a wide range of toxicants apart from those specific to tobacco smoke. These tobacco-related toxicants may come from other sources of exposure besides tobacco smoke. However, their high toxicity makes them worthy of study in different tobacco smoke exposure scenarios in combination with cotinine and other tobacco-specific biomarkers, thus providing a broader perception of the health harms derived from tobacco smoke exposure. The non-specific biomarkers analyzed in the papers reviewed here included polycyclic aromatic hydrocarbons, several kinds of volatile organic compounds, metals and carbon monoxide.

### 5.1. Polycyclic Aromatic Hydrocarbons (PAHs)

Although polycyclic aromatic hydrocarbons (PAHs) are not tobacco smoke-specific markers, they are present in higher concentrations in smoking environments [96]. PAHs are metabolized in the liver by cytochrome P450 generating reactive epoxy intermediates which are converted to its non-reactive hydroxylated forms by epoxide hydrolase. Figure 2B shows the pyrene metabolism as an example of PAH metabolism. To perform body detoxification, PAH intermediaries are conjugated with glucuronide acid or glutathione by UGTs or glutathione S-transferases (GSTs), respectively, and excreted [85,97,98]. As shown in Table 1, half-life time of some common PAH biomarkers vary from 4.1 to 9.4 h [99]. The main health effects of PAHs have been related with immunotoxicity, genotoxicity, cytotoxicity, mutagenicity and carcinogenicity in humans [100]. Short-term exposure to PAHs may also result in several non-carcinogenic effects, such as eye and skin irritation, nausea, vomiting and inflammation. Long-term exposure to PAHs has been related with cataracts, kidney and liver damage, break-down of red blood cells and several types of cancer, such as skin, lung, bladder and gastrointestinal cancer [101]. As an example of the occurrence of PAH biomarkers in relation with tobacco smoke exposure, Kim et al. performed cross-sectional analyses of 1985 children aged 6 to 18 years using data from the 2003–2008 U.S. NHANES survey. SHS exposure, measured as serum cotinine, was strongly associated with urinary concentrations of nine PAH biomarkers, with concentrations in SHS exposed nonsmokers ranging from 118 ng/mL for 1-OHPyr and up to 6046 ng/mL for naphthalene metabolites. PAH biomarkers of non-exposed children were generally lower, as seen in Table 2 [57].

### 5.2. Volatile Organic Compounds (VOCs)

Tobacco smoke also contains a wide range of VOCs, including several carbonyl compounds, such as crotonaldehyde and acrolein, and aromatic compounds like benzene, among others [2]. Exposure to these VOCs is associated with many adverse health effects including irritations, tissue damage, DNA-adducts formation, mutagenicity and even strong carcinogenic effects in the case of benzene [102,103,104]. Following exposure, body detoxification from crotonaldehyde and acrolein mostly begins with the conjugation of glutathione by GSTs in the liver and ends with the production of their main metabolites *N*-acetyl-*S*-(3-hydroxypropyl-1-methyl)-l-cysteine (HPMM) and 3-hydroxypropyl mercapturic acid (HPMA), respectively, which are excreted through urine, as shown in Figure 2B [86,87]. Urinary half-life of HPMM and HPMA is 5–9 h [105]. Waterpipe smoke exposure is a source of acrolein. In waterpipe venues urinary 3-HPMA and cotinine were positively correlated among smokers and nonsmokers with values up to 3686 pmoL/mg cr in daily waterpipe smokers and 2498 pmoL/mg cr in nonsmokers attending a waterpipe social event [68].

Bagchi et al. examined the influence of tobacco exposure and crotonaldehyde in 4692 participants of the 2005–2006 and 2011–2012 NHANES surveys, with mean (IQR) values of 1.63 (068–3.29) in smokers and 0.313 (0.231–0.451) in nonsmokers. Urinary HPMM levels were positively associated with serum cotinine and even though demographic variables, such as age, gender and race, showed distinct effects on crotonaldehyde exposure, authors concluded that tobacco smoke is a major source of crotonaldehyde exposure [55].

An average of 17% of the inhaled benzene is exhaled and the remaining part is metabolized and excreted through urine as unmodified benzene and as other metabolites such as S-phenyl-mercapturic acid (SPMA) or *trans*,*trans*-muconic acid (ttMA) [103,106]. The association between urinary benzene and SHS exposure was measured by Protano et al. in 122 children from an Italian rural area [67]. Benzene median concentrations and IQR for children with smoking parents were 359.5 ± 362 ng/L, whereas non-exposed children’s were 92.5 ± 90 ng/L. For children with smoking parents who did not smoke inside the homes, benzene concentrations were 282 ± 131 ng/L and 314.5 ± 177 ng/L for children whose parents smoked inside the house when children were out, thus indicating the relevance of THS exposure in children. If parents smoked inside when children were in, children’s benzene concentrations were 596 ± 548 ng/L. Urinary cotinine concentrations varied similarly. In a latter study, urinary cotinine was positively correlated with urinary benzene (*r* = 0.164, *p* < 0.05) and its metabolite SPMA (*r* = 0.190, *p* < 0.01) in morning urine samples [107]. Although benzene may occur from different emission sources, the relationship of benzene with SHS and THS was proved in the studied children.

### 5.3. Metals

A numerous amount of toxic metals, such as cadmium and lead, are transferred to tobacco smoke during cigarette burning and absorbed by humans through inhalation. Tobacco smoke is considered to be one of the main source of cadmium and lead intake by humans [108,109]. After absorption, cadmium and lead are transported through the blood to several tissues, such as lungs, kidneys or bones, where they can be accumulated [110,111]. These toxicants are mainly eliminated through urine but their clearance is quite slow (i.e., 0.001% per day of Cd) [108]. As shown in Table 1, half-life time of lead in blood averages 36 days while half-life time of cadmium in urine and blood can be up to one or two decades [110,112]. Both cadmium and lead can produce tubular dysfunction and renal failure in the kidney [113], hence lead is classified as a possible human carcinogen and cadmium as carcinogenic to humans [30].

The presence of heavy metals in combination with cotinine has been assessed in sensible populations. For instance, Polanska et al. reported a mean lead concentration of 1.1 µg/dL with a range from 0.4 to 5.7 µg/dL in cord blood of SHS-exposed newborn babies. Prenatal lead exposure together with a long-term exposure to SHS resulted in a negative effect on the development of motor abilities for children tested in 2-year-olds [22]. In pregnant women at delivery time, Jedrychowski et al. found a low lead concentration of 1.63 µg/dL in whole blood, which might be associated with hypertension during pregnancy. Nevertheless, the occurrence of lead was not clearly correlated with cotinine levels [56]. However, there were small but significant correlations between cotinine and lead in newborns and children DBS [52,114].

The role of SHS exposure in urinary cadmium levels was also not conclusive. As an example, similar cadmium concentrations were found in children exposed and non-exposed to SHS, without clear correlations with urinary cotinine [46,115]. Conversely, Sánchez-Rodríguez et al. found that urinary cadmium levels slightly decreased in 83 adults after the implementation of a restrictive anti-smoking legislation, ranging from 0.17 (0.11–0.29) μg/g cr before the smoking ban to 0.10 (0.06–0.22) μg/g cr one year after the law implementation. The reduction of urinary cotinine was lower than urinary Cd, thus the authors concluded that further monitoring is necessary as Cd variations could be also due to atmospheric Cd exposure and may be influenced by differences in body mass indexes [116]. Nevertheless, a possible correlation of whole blood cadmium concentrations with SHS exposure was studied in 1398 adults participating in the 2007–2012 Korean National Health and Nutrition Examination Survey (KNHANES) that self-reported SHS exposure. Age adjusted blood cadmium levels in adults were higher in nonsmokers exposed to SHS than in non-exposed ones (1.07 µg/L vs. 1.02 µg/L). In addition, blood cadmium levels of both adults and adolescents correlated positively with levels of urinary cotinine [69].

### 5.4. Carbon Monoxide

SHS is considered an important source of exposure to CO in nonsmokers [117]. CO levels in mainstream smoke average 5 to 22 mg/cigarette (approximately 4.5% of tobacco smoke [118]) and are on the level of 9 to 35 mg/cigarette for sidestream smoke [119]. Once released in the atmosphere, CO rapidly diffuses into the body through alveolar, capillary and placental membranes during inhalation. Since CO has 200 to 250 times more affinity to haemoglobin than oxygen, 80% to 90% of CO successfully binds to haemoglobin, forming its main blood metabolite, carboxyhaemoglobin (COHb). As shown in Table 1, half-life time of exhaled CO and blood COHb average 2–6 h and 4–6 h, respectively and therefore they can be used as short-term SHS exposure biomarkers [24]. Cigarette consumption and high concentrations of exhaled CO could be related to low birth weight [120]. However, further research is needed to determine the toxicological importance of CO in SHS exposure. As seen in Table 2, CO concentration ranges between 1.9 and 5.9 ppm in nonsmokers exposed to SHS, whereas in smokers commonly range from 6 ppm (common cutoff value) to 22.81 ppm [47,59]. Exhaled carbon monoxide (CO) from workers who are exposed to SHS in public venues could be measured to investigate indoor air quality [121]. Whole blood COHb correlates with the smoking status, with mean concentrations of 17.57% in smokers and 1.2% in nonsmokers exposed to SHS [70]. Among nonsmokers, 30 min of exposure to waterpipe smoke increased the COHb levels, which correlated with serum cotinine, corroborating the relevance of CO emissions, even in short-time SHS exposure.

## 6. Determination of Multiple Specific Biomarkers. Examples of Applications

In the previous sections we have already commented the suitability of each studied biomarker and their association with cotinine concentrations and the type of tobacco smoke exposure. The aim of this section is to comment some selected examples to further discuss the usefulness of the simultaneous determination of multiple biomarkers for the characterization of either SHS or THS exposure.

### 6.1. Evaluation of SHS Exposure

The intrinsic characteristics of the studied populations have a key role not only in the selection of the biological matrix, but also in the election of biomarkers. For instance, the determination of nicotine in biofluids does not provide very valuable information, the simultaneous analysis of urinary nicotine and cotinine can be suitable in individuals with a decreased ability to metabolize nicotine due to a reduced CYP2A6 activity. In this sense, Matsumoto et al. studied the total nicotine and cotinine urinary concentrations of 117 Japanese nonsmokers, concluding that 54% of these nonsmokers presented higher nicotine concentrations than those found for cotinine [45] and, therefore, the simultaneous determination of both biomarkers provided a better characterization of SHS exposure for that population group.

Although the study of cotinine and 3HC has been extensively used in cohorts of smokers, the simultaneous determination of urinary NNAL and urinary or serum cotinine is usually the preferred approach for characterizing long term SHS. As TSNAs are formed while SHS ages, urinary NNAL/cotinine ratio is 10 times higher in SHS exposure compared to active smoking, without gender, race/ethnicity or age differences [50], and it is estimated to be even higher in young children exposed to THS. Therefore this ratio could be used as a biomarker to distinguish between SHS and THS exposure [6]. The NNAL/cotinine ratio was also higher among pregnant women who did not smoke (0.0076) in comparison to those who smoke (0.0013) [48]. Nevertheless, Benowitz et al. recently suggested that by comparing sensitivity and specificity, the single determination of NNAL has a better performance than the NNAL/cotinine ratio in discriminating smokers from nonsmokers [50].

Since urinary cotinine and NNAL have different metabolic clearances, the joint study of both biomarkers could be useful to evaluate changes in SHS exposure. For instance, the role of SHS exposure in cars was evaluated by exposing eight nonsmoker volunteers to SHS for one hour (3 cigarettes). After this exposure, urinary cotinine increased 6-fold whilst the increase of NNAL was ca. 27-fold in comparison with baseline biomarker levels. In the same study plasma nicotine did not change after SHS exposure [49]. In another example, the application of the smoking ban reduced urinary cotinine and NNAL from mean values of 35.9 ng/mL and 18.2 pg/mL, respectively, to values below the detection limit (<5 ng/mL) for cotinine and 7.3 pg/mL for NNAL, two months after implementing the law [44]. Urinary cotinine and NNAL concentrations also correlate with airborne particulate matter (PM_2.5_) [37,122,123].

Finally, another example of the determination of multiple tobacco smoke biomarkers is the evaluation of SHS exposure in waterpipe venues, which exposure to smoke toxicants may differ from conventional cigarette smoke exposure. For instance, Moon et al. recently investigated the possible correlations of four tobacco specific biomarkers (urinary and salivary cotinine, urinary NNAL and hair cotinine) with two related biomarkers (urinary 1-OHPG and CO in breath). In nonsmoking employees, they found moderate correlations among the tobacco-specific biomarkers, urinary cotinine and 1-OHPG. However, in this study CO concentrations were not associated with any of the tobacco-specific biomarkers studied (salivary cotinine, hair nicotine, urinary NNAL, and exhaled CO) which could be due to the short half-life of CO or the sampling process when business activity was low [36].

### 6.2. Evaluation of THS Exposure

When designing studies to evaluate THS exposure, it is necessary to take into consideration three main characteristics that make THS different from SHS exposure [6]. The first one is that the concentration of TSNAs increases as SHS ages, therefore, the TSNAs/cotinine ratio in nonsmokers exposed to THS is usually higher than in non-exposed ones. The second consideration is that 4-(methylnitrosamino)-4-(3-pyridyl)butanal (NNA) is a TSNA that is specific to THS, and the evaluation of its possible main metabolites—4-(methylnitrosamino)-4-(3-pyridyl)-1-butanol (iso-NNAL) and 4-(methylnitrosamino)-4-(3-pyridyl)butyric acid (iso-NNAC)—would enable the distinction between SHS and THS exposure. However, to date, there is a lack of biomonitoring studies of these specific NNA biomarkers. The third consideration is that unlike SHS, the main pathways of exposure to THS are nondietary ingestion and dermal absorption. Dermal absorption is usually overlooked as a possible pathway of exposure, but it is especially relevant in the case of THS contamination where nonsmokers are exposed to smoke toxicants bound to fabrics, clothing, settle dust and surfaces. Different studies demonstrated that nicotine has a large dermal permeability coefficient (k_p_g_—from air through skin to blood—4.4 m/h) [27], that dermal uptake of nicotine can occur directly from air, which is comparable to the estimated inhalation uptake of nicotine [124] and lastly, that a substantial fraction of tobacco smoke exposure is through dermal absorption [125].

The accumulation of nicotine in the hands of nonsmokers has been proved in several studies. Different research groups of the University of California in San Diego leaded by Matt el al., have measured finger nicotine as a biomarker of THS exposure [42,43]. The main aim of these studies was to examine whether THS persists during long periods of time in different indoor environments. For instance, finger nicotine concentrations of nonsmokers who stayed overnight in guest rooms were up to 17.7 ng/wipe in hotels with complete smoking bans, up to 226.9 ng/wipe in non-smoking rooms in hotels without complete smoking bans and up to 1713.5 ng/wipe in smoking rooms, correlating with the nicotine found on guestroom surfaces and urinary cotinine. Urinary cotinine GM levels were five to six times higher for volunteers staying in smoking rooms. Mean cotinine levels were similar between those volunteers staying in non-smoking rooms of smoke-free and smoking hotels, suggesting that the single study of cotinine did not provide an accurate measure of THS exposure. NNAL was measured only in guests staying in the most polluted smoking rooms. For these guests NNAL’s GM increased from 0.86 pg/mg cr to 1.24 pg/mg cr after staying overnight [43].

In a study published in 2017, homes of former smokers were examined until 6 months after quitting [42]. In the first week after quitting, they observed a significant reduction of nicotine in fingers of non-smoking residents (from 29.1 to 9.1 ng/wipe) without any significant changes thereafter, matching with nicotine levels in surfaces. These findings indicated that surfaces may be the main source of finger nicotine and also that homes of smokers remained polluted with THS for up to 6 months after cessation. Levels of cotinine declined from the first week after quitting, decreasing from a baseline GM concentration of 9.9 to 1.5 ng/mL 1 month after quitting, and remaining stable after that time point. However, NNK exposure declined more gradually since it was not until 3 months after quitting that NNAL levels decreased significantly (11.0 pg/mL at basal level to 3.2 pg/mL 3 months post quitting). After this initial decline, both urinary cotinine and NNAL levels remained stable and above levels found in nonsmokers without exposure to SHS or THS. Authors concluded that smoking cessation did not immediately and completely eliminate exposure risk since homes of smokers remained polluted with nicotine and TSNAs in dust and on surfaces, and residents continued to be exposed for at least 6 months after smoking cessation.

Recently, the relationship between tobacco smoke exposure and hand nicotine was studied in 25 children with potentially tobacco related illnesses [63]. All children had detectable hand nicotine in the range of 18.3 to 690.94 ng/wipe (GM 86.4 ng/wipe) confirming the relevance of THS exposure in young children. Furthermore, hand nicotine levels presented a significant positive association with salivary cotinine, therefore, hand wipes could be useful as a proxy for exposure and to determine overall tobacco smoke pollution. These findings corroborated that tobacco exposure is produced via multiple pathways and, therefore, a comprehensive assessment of tobacco exposure must include both SHS and THS.

Nicotine in fingers has also been used for the assessment of THS transportation, thus Northrup et al. evaluated THS exposure in infants of smoking mothers, admitted to the neonatal intensive care unit on their date of birth [41]. Nicotine in mother’s fingers highly correlated with tobacco biomarkers in infant urine. Levels of urinary NNAL in the newborn were comparable to those levels found in nonsmokers exposed to SHS and to the urinary NNAL concentrations in former smokers just 1 week after quitting [42], thus corroborating the role of THS transportation in tobacco smoke exposure.

Besides, four studies have focused on the suitability of urinary cotinine and NNAL to assess THS exposure. Urinary cotinine values for THS exposure ranged between 0.05 to 5 ng/mL for most of the studies [41,43,44] and were up to 6 ng/mL in one case [42]. These levels exceeded the range proposed by Benowitz et al. of 0.05–0.25 ng/mL for low-level SHS exposure or THS exposure [50]. These values also include cotinine levels of bar employees after a smoking ban implementation and hence, exposed to THS in an environment where smoking was previously permitted [44]. NNAL levels were between 2.7 and 6.7 pg/mL for nonsmokers exposed to THS at home [42] and were up to 7.3 for bar employees exposed to THS at work [123]. These levels have been exceeded in the case of infants admitted in intensive care unit who have smoking mothers (12.4 pg/mL) [41].

## 7. Conclusions

The determination of biomarkers of tobacco smoke exposure plays a key role in the characterization of the health effects related to this exposure. In this review we have discussed the suitability of the determination of multiple biomarkers to assess SHS and THS exposure. The selected biological matrices will determine the kind of information obtained in a concrete study. Urine was the most widely used biological matrix in the studies summarized here, suitable for the assessment of SHS and THS. Nevertheless, depending on the nature of the study, it can be useful to complement the information provided by the urinary biomarkers with the analysis of other biological matrices, such as blood. For short-term exposure, saliva and exhaled breath are commonly studied, whilst hair enables the assessment of long-term exposure. Dermal absorption is usually overlooked as a pathway of exposure to tobacco toxicants, however, several studies have demonstrated the relevance of skin in the transport and accumulation of tobacco smoke toxicants. In the characterization of THS exposure, nicotine in hands and fingers correlated with urinary cotinine and NNAL.

The selection of the appropriate target biomarkers will depend on the available biological matrices (that influence on biomarker availability and half-life time), source of exposure (i.e., cigarettes or waterpipe), objectives of the study (i.e., short term, long-term or intermittent SHS exposure or THS exposure) and the characteristics of the target population (i.e., age, race, specific diseases, etc.). Cotinine is the gold standard biomarker of tobacco exposure. Nevertheless, an approach worthy of further investigation could be the simultaneous determination of urinary cotinine and NNAL that enables a better characterization of low SHS and THS exposure. Besides, NNK and NNN were the most concentrated TSNAs in saliva and hair, respectively, and the identification of these biomarkers in these biomatrices must be considered in future works. The assessment of non-specific biomarkers provided a broader knowledge about the health effects associated with tobacco smoke exposure. Nevertheless, exposure to these biomarkers can also occur from other sources and, therefore, their concentrations are not always clearly linked with tobacco exposure.

Finally, although few studies have focused on determining THS exposure, the data reviewed here confirms the risks of this poorly described tobacco smoke exposure pathway. Consequently, future research must include the assessment of both SHS and THS exposure, especially in the most vulnerable population to THS: Children.

## Figures and Tables

**Table 1:** Biomarkers of tobacco smoke exposure studied in the reviewed papers, their half-life time, precursor toxicant and main toxicological characteristics including: The International Agency for Research on Cancer (IARC) classification; cancer inhalation unit risk, expressed in (µg/m^3^)^−1^; inhalation and oral cancer slope factors, in (mg/kg-day)^−1^; non-cancer chronic inhalation reference exposure level (REL), in µg/m^3^; and other relevant toxicological information. Risk values are from the Office of Environmental Health Hazard Assessment at the California Environmental Protection Agency (OEHHA-CalEPA) Chemical Database [29]. Different sources of information are indicated.

Biomarker	Half-Life Time (t_1/2_) ^a^	Toxicant Precursor	IARC Classification ^b^	Cancer		Non-Cancer	Other
				Inhalation Unit Risk	Slope Factor	Chronic Inhalation REL	
Tobacco smoke specific biomarkers							
Nicotine	Blood (t_1/2_): 2 h Urine (t_1/2_): 11 h	Nicotine	NA	NA	NA	NA	Reproductive toxicity
Cotinine	Saliva (t_1/2_): 15 h Blood (t_1/2_): 16 h Urine (t_1/2_): 3–4 days						
*trans*-3’-hydroxycotinine (3HC)	Blood (t_1/2_): 6.6 h Urine (t_1/2_): 6.4 h						
N′-nitrosonornicotine (NNN)	NA	NNN	1	4.0 × 10^−4^	1.4	NA	NSRL: 0.5 µg/day
4-(methylnitrosoamino)-1-(3-pyridyl)-1-butanone (NNK)	Urine (t_1/2_): 2.6 h	NNK	1	5.2 × 10^−3 c^	49 (oral)	NA	NSRL: 0.014 µg/day
4-(methylnitrosoamino)-1-(3-pyridyl)-1-butanol (NNAL)	Urine (t_1/2_): 40–45 days						
Tobacco smoke related biomarkers							
Polycyclic aromatic hydrocarbon biomarkers							
1-hydroxy naphthalene (1-OHNap) 2-hydroxy naphthalene (2-OHNap)	NA	Naphthalene	2B	3.4 × 10^−5^	0.12	9	NSRL: 5.8 µg/day
	Urine (t_1/2_): 9.4 h						
2-hydroxy fluorene (2-OHFlu) 3-hydroxy fluorene (3-OHFlu) 9-hydroxy fluorene (9-OHFlu)	Urine (t_1/2_): 4.1 h	Fluorene	3	NA	NA	NA	NA
	NA						
	NA						
1-hydroxy phenanthrene (1-OHPA) 2-hydroxy phenanthrene (2-OHPA) 3-hydroxy phenanthrene (3-OHPA)	NA	Phenanthrene	3	NA	NA	NA	NA
	NA						
	NA						
1-hydroxy-pyrene (1-OHPyr) 1-hydroxy-pyrene glucuronide (1-OHPyrG)	Urine (t_1/2_): 6 h NA	Pyrene	3	NA	NA	NA	NA
Volatile organic compounds biomarkers							
Benzene	NA	Benzene	1	2.9 × 10^−5^	0.1	3	Reproductive toxicity NSRL: 13 (inhalation) 6.4 (oral) µg/day
N-acetyl-S-(3-hydroxypropyl-1-methyl)-L-cysteine (HPMM)	Urine (t_1/2_): 5–9 h	Crotonaldehyde	3	NA	1.9 (oral) ^d^	NA	NA
3-hydroxypropyl mercapturic acid (HPMA)	Urine (t_1/2_): 5–9 h	Acrolein	3	NA	NA	0.35	NA
Metals							
Cadmium	Blood and urine (t_1/2_): 1–2 decades	Cadmium	1	4.2 × 10^−3^	15	0.02	Reproductive toxicity NSRL (inhalation): 0.05 µg/day
Lead	Blood (t_1/2_): 36 days	Lead	2B	1.2 × 10^−5^	0.042 (inhalation) 8.5 × 10^−3^ (oral)	NA	Reproductive toxicity NSRL (oral): 15 µg/day
Other							
Carbon monoxide (CO) Carboxyhemoglobin (COHb)	Exhaled (t_1/2_): 2–6 h	CO	NA	NA	NA	23,000 (acute REL)	Reproductive toxicity
	Blood (t_1/2_): 4–6 h						

^a^ Half-life time references of each metabolite are described in Section 4 and Section 5. ^b^ IARC classification: 1—carcinogenic to humans—; 2B—possibly carcinogenic to humans—; 3—not classifiable as to its carcinogenicity to humans [30]. ^c^ Information from Naufal et al. [31]. ^d^ Data from the Risk Assessment Information System (RAIS) [32]. Glossary: NSRL (No significant risk level): Daily intake level posing a 10^−5^ lifetime risk of cancer [29]; Chronic Inhalation non-cancer REL (Reference exposure level): Concentration level at or below which no adverse health effects are anticipated for a specified exposure duration [33]; Cancer slope factor: Toxicity value for evaluating the probability of an individual developing cancer from exposure to contaminant levels over a lifetime [32]; Unit risk (UR): Estimation of the increased cancer risk from the exposure to a concentration of 1 µg/m^3^ for a lifetime [34]. NA: Not available.

**Table 2:** Summary of the most common concentration ranges of the studied biomarkers in nonsmokers, accordingly with the reviewed references. Concentration ranges have been classified regarding the type of tobacco smoke exposure of the target population: “Smokers” for the smoker population and “SHS exposure”, “THS exposure” or “No exposure”, for non-exposed population.

Biomarker	Matrix	Smokers	SHS Exposure	THS Exposure	No Exposure	References
Nicotine	Hair	2.01–79.30 ng/mg (min–max)	0.08–5.02 ng/mg (IQR)	NA	NA	[23,35,36,37,38,39,40]
	Skin	44–1160 ng/wipe (min–max)	25.6 (13.2–48.9) ng/wipe (GM (95% CI))	2.9 (<LOD–46.1) ng/wipe (GM (95% CI)	2.5 (<LOD–17.7) ng/wipe (GM (min–max))	[41,42,43]
Cotinine	Urine	34.5–489.15 ng/mL (GM range)	0.25–30 ng/mL (min–cutoff point)	0.05–5 ng/mL (Cutoff range)	0.88 ng/mL (max value)	[22,36,41,42,43,44,45,46,47,48,49,50]
	Serum/Plasma	>10–499 ng/mL (cutoff–max)	0.015–14.6 ng/mL (Cutoff range)	NA	<LOD (<0.05) ng/mL	[49,51,52,53,54,55,56,57,58]
	Saliva	>13–653 ng/mL (cutoff–IQR)	0.04–14.9 ng/mL (min–max)	NA	NA	[36,38,39,40,59,60,61,62,63]
	Hair (min–max)	0.08–2.49 ng/mg	0.05–1.57 ng/mg	NA	NA	[23,35,64]
3HC	Urine (Mean (SD))	653.81 (62.30) µg/g cr	60.79 (46.70) µg/g cr	NA	NA	[65]
NNN	Saliva (Mean (IQR))	118 (3.9–91) pg/mL	5.3 (1.2–2.9) pg/mL ^a^	NA	NA	[60]
NNK	Saliva (Mean (IQR))	6.6 (2.8–7.1) pg/mL	4.5 (2.4–5.2) pg/mL ^a^	NA	NA	[60]
NNAL	Urine	80.9–405.5 pg/mL (Median range)	Low: 0.95–2.21 pg/mL (GM Range) High: 5.9–20.1 pg/mL (*CI*)	2.7–6.7 pg/mL (GM range)	0.86 pg/mg cr (CI)	[36,42,43,44,48,50,51,54,58,61,66]
	Saliva (Mean (IQR))	3.2 (0.98–3.5) pg/mL	1.3 (0.83–1.8) pg/mL ^a^	NA	NA	[60]
1-OHNap, 2-OHNap	Urine	NA	4587.6–6045.6 ng/L ^b^	NA	4466.1 ng/L (GM)	[57]
2-OHFLu, 3-OHFlu, 9-OHFlu	Urine	NA	571.0–824.8 ng/L ^b^	NA	439.9 ng/L (GM)	[57]
1-OHPA, 2-OHPA, 3-OHPA	Urine	NA	288.1–351.2 ng/L ^b^	NA	241.2 ng/L (GM)	[57]
1-OHPyr	Urine	NA	118.1–165.1 ng/L ^b^	NA	95.7 ng/L (GM)	[57]
Benzene	Urine (Median ± IQR)	NA	596 ± 548 ng/L	Low: 282 ± 131 ng/L High: 314.5 ± 177 ng/L	92.5 ± 90 ng/L	[67]
HPMM	Urine (Median (IQR))	1.63 (0.680–3.29) mg/g cr	NA	NA	0.313 (0.231–0.451) mg/g cr	[55]
HMPA	Urine (IQR)	1203–4898 pmol/mg cr	1580–3964 pmol/mg cr	NA	NA	[68]
Cadmium	Urine (CI)	NA	0.11–0.29 µg/L	NA	0.097–0.12 μg/L	[46]
	Whole blood ^c^	NA	1.07 µg/L	NA	1.02 µg/L	[69]
CO	Exhaled breath	>6–22.81 ppm (cutoff–mean)	1.9–5.9 ppm (min–max)	NA	NA	[47,59]
COHb	Plasma (Mean (SD))	17.57% (8.79)	1.2% (0.8)	NA	NA	[70]

^a^: Tobacco smoke exposure not specified; ^b^: GM range between low and high SHS exposure; ^c^: Age adjusted blood cadmium level. CI: Confidence interval; GM: Geometric mean; IQR: Interquartile range; LOD: Limit of detection; SD: Standard deviation. NA: Not available.

3HC: *trans*-3’-hydroxycotinine; NNN: N′ -nitrosonornicotine; NNK: 4-(methylnitrosoamino)-1-(3-pyridyl)-1-butanone; NNAL: 4-(methylnitrosoamino)-1-(3-pyridyl)-1-butanol; 1-OHNap: 1-hydroxy naphthalene; 2-OHNap: 2-hydroxy naphthalene; 2-OHFLu: 2-hydroxy fluorene; 3-OHFlu: 3-hydroxy fluorene; 9-OHFlu: 9-hydroxy fluorene; 1-OHPA: 1-hydroxy phenanthrene; 2-OHPA: 2-hydroxy phenanthrene; 3-OHPA: 3-hydroxy phenanthrene; 1-OHPyr: 1-hydroxy-pyrene; HPMM: N-acetyl-S-(3-hydroxypropyl-1-methyl)-L-cysteine; HMPA: 3-hydroxypropyl mercapturic acid; CO: carbon monoxide; COHb: carboxyhemoglobin.

## Supplementary Materials

The following are available online at http://www.mdpi.com/1660-4601/15/12/2693/s1. Table S1: Summary of the results obtained in the papers included in this review. The data presented in this table corresponds to the biomarker concentrations from the nonsmokers studied that were exposed to second-hand (SHS) or thirdhand smoke (THS), including the main characteristics of the target population, the analyzed biomatrix and the biomarker concentration, expressed as reported in the original paper. The smokers (S) concentrations are also summarized, if available.
