# Supplementary material for: Biomarkers of Exposure to Secondhand and Thirdhand Tobacco Smoke: Recent Advances and Future Perspectives
Source: Int J Environ Res Public Health. 2018 Nov 29;15(12):2693. doi: 10.3390/ijerph15122693 (PMC6313747; doi:10.3390/ijerph15122693)
Supplement: Supplementary file 1 [file ijerph-15-02693-s001.pdf]

Supplementary Information

Table S1. Summary of the results obtained in the papers included in this review. The data presented in this table corresponds to the biomarker concentrations from the nonsmokers studied that were exposed to sencondhand *(SHS)* or thirdhand smoke *(THS)*, including the main characteristics of the target population, the analyzed biomatrix and the biomarker concentration, expressed as reported in the original paper. The smokers *(S)* concentrations are also summarized, if available.

Reference	Target population	Biological matrix	Biomarkers	Concentrations
[1]	Pregnant women: n= 431, age: 18-35 years<br>Low SHS: Low exposure to $PM_{2.5}$<br>High SHS: High exposure to $PM_{2.5}$<br>(Poland)	Serum	Cotinine<br>*Not specified*	*Low SHS:* 0.16 ng/mL<br>*High SHS:* 0.33 ng/mL
		Whole blood	Lead<br>*GM (95% CI)*	*SHS:* 1.63 (1.63-1.75) µg/dL
[2]	Children: n= 122, age: 5-11 years, male and female<br>Exposure to SHS and THS at home<br>Low THS: If the cohabitants smoke outside the house<br>High THS: If the cohabitants smoke inside the house when children are out<br>(Italy)	Urine	Cotinine<br>*Median ±IQR*	*Low THS:* 3.04 ± 4.19 µg/g cr<br>*High THS:* 3.61 ± 21.92 µg/g cr<br>*SHS:* 5.95 ± 51.23 µg/g cr
			Benzene<br>*Median ±IQR*	*Low THS:* 282 ± 131 ng/L<br>*High THS:* 314.5 ± 177 ng/L<br>*SHS:* 596 ± 548 ng/L
[3]	Adults: n= 27-23, age: 21-37 years, male and female<br>3 hours of controlled SHS exposure in a terrace of a bar or a restaurant (Rest.)<br>(USA)	Urine	NNAL<br>*GM (min-max)*	*Bar (SHS):* 0.109 (0-3.60) pg/mL<br>*Rest. (SHS):* 0.008 (0-2.1) pg/mL
		Saliva	Cotinine<br>*GM (min-max)*	*Bar (SHS):* 0.161 (0.094-0.407) ng/mL<br>*Rest. (SHS):* 0.075 (0.036-0.188) ng/mL
[4]	Adults (A): Smokers n = 25, SHS exposed n = 19<br>Children (C) exposed to SHS: n = 18, age: ≤ 2years<br>(Greece)	Hair	Cotinine<br>*A: Mean (min-max)*<br>*C: Min-max*	*A (S):* 1.16 (0.08-2.49) ng/mg
				A (SHS): 0.13 (0.05-0.32) ng/mg<br>*C (SHS):* 0.13-1.57 ng/mg
			Nicotine<br>*A: Mean (min-max)*<br>*C: min-max*	*A (S):* 27.97 (2.01-79.30) ng/mg
				A (SHS): 1.49 (0.12-2.58) ng/mg<br>*C (SHS):* 0.69-28.71 ng/mg
[5]	Bar employees: n= 40, age: 21-73 years, 70% female<br>Exposure before smoking ban (Bef.) and after ban (Aft.)<br>(USA)	Urine	Cotinine<br>*Mean (SD)*	*Bef. (SHS):* 35.9 (17.4) ng/mL<br>*Aft.:* Non-quantifiable (<5 ng/mL)
			NNAL<br>*Mean (SD)*	*Bef. (SHS):* 0.087 (0.065) pmol/mL<br>*Aft.:* 0.035 (0.033) pmol/mL
[6]	Bar and restaurant employees: Smokers n = 21, SHS exposed n = 17, age: <25-≥35 years, male and female.	Urine	Cotinine<br>*Mean (SD)*	*Bef. (S):* 67.26 (0.86) ng/mL<br>*Aft. (S):* 63.16 (10.81) ng/mL

	Before smoking ban (Bef.) and after ban (Aft.)<br>(Ankara, Turkey)			*Bef. (SHS):* 61.24 (4.66) ng/mL<br>*Aft. :* 56.26 (7.66) ng/mL
		Breath	CO<br>*Mean (SD)*	*Bef. (S):* 22.81 (12.23) ppm<br>*Aft. (S):* 14.29 (7.41) ppm
				Bef. (SHS): 3.94 (5.26) ppm<br>*Aft.:* 1.82 (1.98) ppm
[7]	Adults with self-reported asthma: Smokers and Nonsmokers with possible SHS exposure (NS) n = 456, age: 20->65 years, male and female.<br>NHANES<br>(USA)	Serum	Cotinine<br>*Median (min-max)*	*S:* 265.5 (87-499) ng/mL
				NS: 0.048 (0.01-57) ng/mL
		Urine	NNAL<br>*Median (min-max)*	*S:* 405.5 (49.6-1770) pg/mL
				NS: 0.55 (0.4-223) pg/mL
[8]	Children: n= 1541, age: 7-48 months, male and female. Assessment of SHS in DBS collected from children for lead screening.<br>(USA)	Dried blood spots (DBS)	Cotinine<br>*Median range*	*NS:* <0.3-5.06 ng/g
			Lead<br>*Min-max*	*NS:* <1-9 µg/dL
[9]	Adults: Smokers n = 815-2146, SHS exposed n = 1651-1891 age: 18-99 years, male and female<br>(UK)	Saliva	Cotinine<br>*S: cutoff*<br>*SHS: Min-max*	*S:* > 15 ng/mL
				SHS: 0.1-14.9 ng/mL
		Breath	CO<br>*S: cutoff*<br>*SHS: Min-max*	*S:* > 6 ppm
				SHS: 1.9-5.9 ppm
[10]	Preschool children: n= 329, age: 2-7 years, 45.9% female<br>(China)	Urine	Cotinine<br>*Mean (SD)*	*SHS:* 1.02 (0.56) log-transformed urinary cotinine-to-cr ratio
			Cadmium<br>*Median (IQR)*	*SHS:* 0.28 (0.21-0.37) nmol/mmol Cd-to-cr
[11]	Adults (A): Smokers n = 83, SHS exposed n = 99, age: 18-80 years<br>Children (C): 283, age: ≤4-10 years, male and female<br>(USA)<br>**Subjects with a mean plasma cotinine > 10 ng/mL (n=81).*	Dried blood spots (DBS) or plasma	Cotinine<br>*A: min-max*<br>*C: Median (min-max)*	*A (S):* 0.9-443 ng/mL *(plasma)*
				A (SHS): <0.02-56 ng/mL *(plasma)*<br>*C (NS):* 0.42 (<0.3-55) ng/g *(DBS)*
			3HC<br>*min-max*	*A (S)*:* 0.2-183 ng/mL *(plasma)*<br>*A (S)*:* 0.4-42 ng/g *(DBS)*
			Lead<br>*min-max*	*C (NS):* <2-9 µg/dL *(DBS)*
[12]	Pregnant women (A)*: n= 55–384, mean age= 29.8 years<br>Children (C): n= 116-233, age = birth, 1 and 2 years, 52.7 % female<br>Polish Mother and Child Cohort Study	Urine	Cotinine<br>*Mean (min-max)*	*C (NS)*<br>*1 year:* 7.1 (<LOD-66.7) ng/mL<br>*2 years:* 8.1 (<LOD-32.3) ng/mL
			1-OHPyr<br>*Mean (min-max)*	*A*:* 0.5 (0.01-8.5) µg/g cr

	(Poland)<br><br>*Possible smokers*	Cord blood	Lead<br>*Mean (min-max)*	C *(NS)*<br>*Birth:* 1.1 (0.4-5.7) µg/dL
		Hair	Mercury<br>*Mean (min-max)*	*A*:* 0.3 (0.02-1.5) µg/g
		Saliva	Cotinine<br>*Min-max*	*A*:* <LOD – 400 ng/mL
[13]	Hospitality venue employees: n = 35-44, age: 18-65 years<br>Before smoking ban (Bef.) and after ban (Aft.)<br>(Switzerland)	Saliva	Cotinine<br>*Mean (95% CI)*	*Bef. (SHS):* 0.67 (0.04-1.30) ng/mL<br>*Aft. 3-6 months:* 2.75 (0.32-5.17) ng/mL<br>*Aft. 9-12 months:* 0.81 (0.00-1.61) ng/mL
			Nicotine<br>*Mean (95% CI)*	*Bef. (SHS):* 1.99 (0.98-3.00) ng/mL<br>*Aft. 3-6 months:* 2.42 (0.01-4.86) ng/mL<br>*Aft. 9-12 months A:* 2.81 (0.12-5.75) ng/mL
[14]	Adults: n = 26, age: 18-50 years, 15 female<br>30 min exposure to tobacco smoke.<br>AE: After exposure<br>BB: Before bed<br>1stAM: 1st moorning<br>22H: 22hours after exposure<br>(USA)	Urine	Cotinine<br>*Median (IQR)*	*AE (SHS):* 0.98 (0.37-1.48) ng/mg cr<br>*BB:* 2.13 (1.55-3.35) ng/mg cr<br>*1st AM:* 1.80 (1.26-3.29) ng/mg cr<br>*22H:* 1.26 (1.10-1.87) ng/mg cr
			3HC<br>*Median (IQR)*	*AE (SHS):* 1.34 (0.85-2.05) ng/mg cr<br>*BB:* 6.29 (4.58-9.36) ng/mg cr<br>*1stAM:* 7.55 (5.13-10.37) ng/mg cr<br>*22H:* 7.74 (5.41-12.99) ng/mg cr
			NNAL<br>*Median (IQR)*	*AE (SHS):* 4.45 (2.70-6.75) pg/mg cr<br>*BB:* 3.99 (2.54-5.53) pg/mg cr<br>*1stAM:* 2.73 (2.08-3.87) pg/mg cr<br>*22H:* 2.44 (1.60 -3.52) pg/mg cr
[15]	Adults: Smokers n = 40, SHS exposed n = 25, mean age: 20.6 years, male and female<br>Silesian Medical University<br>(Poland)	Urine	Cotinine<br>*Mean (SD)*	*S:* 523.10 (68.10) µg/g cr
				SHS: 40.89 (24.80) µg/g cr
			OH-Cot<br>*Mean (SD)*	*S:* 653.81 (62.30) µg/g cr
				SHS: 60.79 (46.70) µg/g cr
[16]	Pregnant women: Smokers n = 57, SHS exposed n = 60-367<br>Low SHS: ≤2 sources of exposure<br>High SHS: >2 sources of exposure<br>Rhea, Mother Childbirth Cohort<br>(Greece)	Urine	Cotinine (n=367)<br>*GM (95% CI)*	*Low SHS:* 7.6 (6.45-8.76) ng/mL<br>*High SHS:* 15.61 (13.03-18.2) ng/mL
			NNAL (n=60)<br>*GM (95% CI)*	*S:* 0.612 (0.365-0.860) pmol/mL
				Low SHS: 0.049 (0.038-0.060) pmol/mL<br>*High SHS:* 0.072 (0.055-0.089) pmol/mL
[17]	Adults: Smokers n = 102, Nonsmokers with possible SHS exposure (NS) n = 117, age: 18-60 years, male and female<br>(Japan)	Urine	Nicotine<br>*Median (IQ)*	*S*: 1635.2 (2222.2) ng/mL
				NS: 3.5 (5.3) ng/mL
			Cotinine<br>*Median (IQ)*	*S:* 3948.1 (3512.2) ng/mL
				NS: 2.8 (4.2) ng/mL

[18]	Adults: Smokers n = 107 (BL) and n = 18 (Follow), SHS exposed n = 105 (BL) and n = 34 (Follow), age: 27-52 years, male and female<br>BL: Baseline<br>Follow: After 2 months follow-up<br>(USA)	Saliva	Cotinine<br>*Median (IQR)*	*BL (S):* 181.0 (76.3-290.2) ng/mL<br>*Follow (S):* 135.1 (62.2-228.6) ng/mL
				BL (SHS): 0.27 (0.04-0.80) ng/mL (26% < LOD)<br>*Follow (SHS):* 0.41(0.035-1.08) ng/mL (27% < LOD)
		Hair	Nicotine<br>*Median (IQR)*	*BL (S):* 16.2 (4.0-40.6) ng/mg<br>*Follow (S):* 16.4 (3.3-27.3) ng/mg
				BL (SHS): 0.36 (0.17-3.03) ng/mg (52% < LOD)<br>*Follow (SHS):* 0.29 (0.20-3.30) ng/mg (59% < LOD)
[19]	Adults: n = 8, age: 18-34 years, 4 females<br>Before (BE) and after (AE) exposure to 1h of SHS exposure in a vehicle<br>(USA)	Plasma	Cotinine<br>*Average (SD)*	*BE:* 0.04 (0.03) ng/mL<br>*AE (SHS):* 0.17 (0.05) ng/mL
		Urine	Cotinine<br>*Average (SD)*	*BE:* 0.38 (0.25) ng/mg cr<br>*AE (SHS):* 2.41 (1.79) ng/mg cr
			Oh-Cot + Cotinine<br>*Average (SD)*	*BE:* 0.006 (0.005) nmol/mg cr<br>*AE (SHS):* 0.025 (0.020) nmol/mg cr
			NNAL<br>*Average (SD)*	*BE:* 0.10 (0.19) pg/mg cr<br>*AE (SHS):* 2.68 (1.36) pg/mg cr
[20]	Adults: Smokers n = 47, SHS exposed n = 15, mean age: 24.9 years, male and female<br>Before (BE) and after (AE) smoking or 30 min of being exposed to water pipe smoke<br>(Israel)	Plasma	Nicotine<br>*Mean (SD)*	*BE (S):* 1.1 (4.1) ng/mL<br>*AE (S):* 19.1 (13.9) ng/mL
				BE (SHS): 0.44 (1.7) ng/mL<br>*AE (SHS):* 0.4 (1.4) ng/mL
			Cotinine<br>*Mean (SD)*	*BE (S):* 61.2 (96.7) ng/mL<br>*AE (S):* 78.2 (93.7) ng/mL
				BE (SHS): 9.2 (25) ng/mL<br>*AE (SHS):* 13.9 (46) ng/mL
		Urine	Nicotine<br>*Mean (SD)*	*BE (S):* 70.4 (232.2) ng/mL<br>*AE (S):* 290.8 (319.6) ng/mL
			Cotinine<br>*Mean (SD)*	*BE (S):* 146.2 (232.2) ng/mL<br>*AE (S):* 165.3 (243.6) ng/mL
		Whole blood	COHb<br>*Mean (SD)*	*BE (S):* 2.02% (2.89)<br>*AE (S):* 17.57% (8.79)
				BE (SHS): 0.8% (0.2)<br>*AE (SHS):* 1.2% (0.8)
[21]	Children: n = 1985, age: 6-18 years, 49.4% female.<br>NHANES<br>(USA)	Serum	Cotinine<br>*Cut-off range*	*Low SHS*: 0.015-0.1 ng/mL<br>*Medium SHS*: 0.1-1.0 ng/mL<br>*High SHS*: 1.0-10.0 ng/mL

		Urine	1-OHNap, 2-OHNap<br>*GM*	*Low SHS*: 4587.6 ng/L<br>*Medium SHS*: 5439.8 ng/L<br>*High SHS*: 6045.6 ng/L
			2-OHFLu, 3-OHFlu, 9-OHFlu<br>*GM*	*Low SHS*: 571.0 ng/L<br>*Medium SHS*: 677.2 ng/L<br>*High SHS*: 824.8 ng/L
			1-OHPA,2-OHPA, 3-OHPA<br>*GM*	*Low SHS*: 288.1 ng/L<br>*Medium SHS*: 352.4 ng/L<br>*High SHS*: 351.2 ng/L
			1-OHPyr<br>*GM*	*Low SHS*: 118.1 ng/L<br>*Medium SHS*: 139.6 ng/L<br>*High SHS*: 165.1 ng/L
[22]	Adults: n = 10 – 30 rooms<br>Low THS: Overnight in nonsmoking rooms of hotels without smoking ban n = 30<br>High THS: Overnight in smoking rooms of hotels without smoking ban n = 29<br>NE: Not exposure - Overnight in hotels with complete smoking ban n = 9<br>BE: Before overnight exposure<br>AE: After overnight exposure<br>(USA)	Finger	Nicotine<br>*Median (min-max)*	*Low THS:* 13.6 (0-226.9) ng/wipe<br>*High THS:* 93.7 (0-1713.5) ng/wipe<br>*NE*: 2.5 (0-17.7) ng/wipe
		Urine	Cotinine<br>*Median (min-max)*	*Low THS:* 0.10 (0-0.41) ng/mL<br>*High THS:* 0.64 (0-2.64) ng/mL<br>*NE:* 0.05 (0-0.88) ng/mL
			NNAL<br>*GM*<br>(10 most polluted smoking rooms)	*BE*: 0.86 pg/mg cr<br>*AE (THS)*: 1.24 pg/mg cr
[23]	Adults: n = 83, age: 25-62 years, 59 female<br>Before smoking ban (Bef.) in 2010 and after ban (Aft.) in 2011<br>(Spain)	Urine	Cotinine<br>*Median (p25,p50)*	*Bef. (S):* 839.8 (318.0-1167.2) μg/g cr<br>*Aft. (S):* 865.7 (443.2-1262.2) μg/g cr
				Bef. (SHS): 0.8 (0.5-1.2) μg/g cr<br>*Aft.:* 0.7 (0.4-1.0) μg/g cr
			Cadmium<br>*Median (p25,p50)*	*Bef. (S):* 0.25 (0.17-0.43) μg/g cr<br>*Aft. (S):* 0.24 (0.17-0.38) μg/g cr
				Bef. (SHS): 0.17 (0.11-0.29) μg/g cr<br>*Aft.:* 0.10 (0.06-0.22) μg/g cr
[24]	Adults: Smokers n = 404-420, SHS exposed n = 116-118, age: >20 years, male and female<br>NHANES<br>(USA)	Serum	Cotinine<br>*GM (95%CI)*	*S*: 149.205 (133.602-166.63) ng/mL
				SHS: 0.717 (0.254-2.022) ng/mL
		Urine	NNAL<br>*GM (95%CI)*	*S*: 0.212 (0.184-0.246) ng/mL
				SHS: 0.0109 (0.0059-0.0201) ng/mL
[25]	Staff in pub and restaurants (Rest.): n = 101, mean age: 47.4 years, 69% female<br>Sampling based on the type (Rest. and Pubs) and size (<150 m^2 and ≥150 m^2) of facilities	Urine	Cotinine<br>*GM (GSD)*	Rest. < 150 m^2<br>*Bef. (SHS)*: 1.5 (2.0) ng/mg cr<br>*Aft.*: 1.9 (2.4) ng/mg cr<br>Rest. ≥ 150 m^2

	Before smoking ban (Bef.) and after ban (Aft.)<br>(Korea)			*Bef. (SHS)*: 1.3 (1.9) ng/mg cr<br>*Aft.*: 1.4 (2.4) ng/mg cr<br>Pub < 150 m^2<br>*Bef. (SHS)*: 2.6 (3.1) ng/mg cr<br>*Aft.*: 3.5 (3.1) ng/mg cr<br>Pub ≥ 150 m^2<br>*Bef. (SHS)*: 1.9 (2.9) ng/mg cr<br>*Aft.*: 3.0 (4.1) ng/mg cr
			NNAL<br>*GM (GSD)*	Rest. < 150 m^2<br>*Bef. (SHS)*: 6.4 (1.9) pg/mg cr<br>*Aft.*: 6.2 (2.1) pg/mg cr<br>Rest. ≥ 150 m^2<br>*Bef. (SHS)*:4.3 (2.3) pg/mg cr<br>*Aft.*: 4.9 (2.1) pg/mg cr<br>Pub <150 m^2<br>*Bef. (SHS)*: 9.8 (2.3) pg/mg cr<br>*Aft.*: 10.4 (2.3) pg/mg cr<br>Pub ≥ 150 m^2<br>*Bef. (SHS)*: 12.1 (2.0) pg/mg cr<br>*Aft.*: 7.3 (1.7) pg/mg cr
[26]	Adults: Smokers n = 166 (quantificable: 70-163), Nonsmokers with possible SHS exposure (NS) n = 529 (quantificable: 8-81), female median age: 58 years, male median age: 55 years, 54% female<br>(Spain)	Oral Fluid	Cotinine<br>*Median (IQR)*	*S*: 358 (74-653) ng/mL<br>*NS*: 0.17 (0.13-0.31) ng/mL
			NNN<br>*Median (IQR)*	*S*: 17 (3.9-91) pg/mL<br>*NS*: 1.6 (1.2-2.9) pg/mL
			NNK<br>*Median (IQR)*	*S*: 4.0 (2.8-7.1) pg/mL<br>*NS*: 3.0 (2.4-5.2) pg/mL
			NNAL<br>*Median (IQR)*	*S*: 1.7 (0.98-3.5) pg/mL<br>*NS*: 1.2 (0.83-1.8) pg/mL
[27]	Newborns (C) in ICU with smoker mothers (A)<br>n = 5<br>(USA)	Finger	Nicotine<br>*min-max*	*A (S)*: 44-1160 ng/wipe
		Urine	Cotinine<br>*min-max*	*C (THS)*: 0.17-5.01 ng/mL
			OH-Cot<br>*min-max*	*C (THS)*: 0.63-31.58 ng/mL
			NNAL<br>*min-max*	*C (THS)*: 0.47-12.38 pg/mL
[28]	Employees exposed to SHS in public places:<br>GB: Government buildings n = 10	Urine	Cotinine<br>*GM*	*GB (SHS):* 4.99 µg/g cr<br>*LB (SHS):* 1.00 µg/g cr

	LB: Large Buildings n = 11<br>N: Nurseries n = 6<br>EI: Private educational institutions n=8<br>(Korea)			*N (SHS):* 0.78 µg/g cr<br>*EI (SHS):* 5.28 µg/g cr
		Hair	Nicotine<br>*GM*	*GB (SHS):* 0.86 ng/mg<br>*LB (SHS):* 0.47 ng/mg<br>*N (SHS):* 0.14 ng/mg<br>*EI (SHS):* 1.62 ng/mg
		Urine	NNAL<br>*GM*	*GB (SHS):* 2.93 µg/g cr<br>*LB (SHS):* 0.84 µg/g cr<br>*N (SHS):* 0.34 µg/g cr<br>*EI (SHS):* 1.84 µg/g cr
[29]	Adults: n = 20, age: >18 years, 9 female<br>SHS exposed at home and/or hopatility venues<br>(USA)	Hair	Nicotine<br>*Median (min-max)*	*SHS:* 0.1 (0-5.5) ng/mg
		Saliva	Cotinine<br>*Min-max*<br>*Cut-off value*	*SHS:* 0 - 11 ng/mL<br>*Cutoff low SHS:* < 10 ng/mL<br>*Cutoff high SHS:* > 10 ng/mL - 13 ng/mL
[30]	Adults: SHS exposed at home n = 24, Non-exposed at home (NE) n = 24, male and female<br>(Spain)	Hair	Nicotine<br>*Median (IQR)*	*SHS*: 2040 (1200-4650) pg/mg<br>*NE*: 623 (221-1160) pg/mg
			Cotinine<br>*Median (IQR)*	*SHS*: 49 (26-106) pg/mg<br>*NE*: 26 (16-43) pg/mg
			NNN<br>*Median (IQR)*	*SHS*: 0.54 (0.29-0.60) pg/mg<br>*NE*: 0.41 (NO IQR) pg/mg
			NNK<br>*Median (IQR)*	*SHS*: 1.3 (0.92-2.7) pg/mg
				NE: 0.74 (0.31-1.1) pg/mg
			NNAL	*SHS*: Not quantified
				NE: Not quantified
[31]	Children: n = 559, age : 12-19 years old, 48.2% female<br>NHANES<br>(USA)	Serum	Cotinine<br>*Cutoff range*	*Low SHS*: 0.05-0.268 ng/mL<br>*High SHS*: 0.268-14.6 ng/mL
		Urine	NNAL<br>*Cutoff range*	*Low SHS* : 0.001-0.005 ng/mL cr<br>*High SHS*: ≥0.005-≤0.082 ng/mL cr
[32]	Adults: n = 6-25, mean age: 49 years, 51% female<br>Non smokers living with former smokers<br>BL: Baseline n = 9-25<br>W1: Week 1 postquit n = 9-17<br>M1: Month 1 postquit n = 9<br>M3: Month 3 postquit n = 8<br>M6: Month 6 postquit n = 6<br>(USA)	Finger	Nicotine<br>*GM (95% CI)*	*BL (SHS):* 25.6 (13.2-48.9) ng/wipe<br>*W1 (THS):* 9.1 (3.8-20.2)ng/wipe<br>*M1 (THS):* 10.2 (4.7-21.2) ng/wipe<br>*M3 (THS):* 5.2 (0.9-19.9)ng/wipe<br>*M6 (THS):* 2.9 (0-46.1) ng/wipe
		Urine	Cotinine<br>*GM (95% CI)*	*BL (SHS):* 9.9 (2.6-32.5) ng/mL<br>*W1 (THS):* 6.0 (1.2-20.8) ng/mL<br>*M1 (THS):* 1.5 (0.5-3.4) ng/mL<br>*M3 (THS):* 1.7 (0.4-4.2)ng/mL

				M6 (THS): 2.7 (0-6.3) ng/mL
			NNAL<br>*GM (95% CI)*	*BL (SHS):* 10.7 (4.7-24.2) pg/mL<br>*W1 (THS):* 6.7 (2.5-15.9) pg/mL<br>*M1 (THS):* 6.7 (3.5-12.2) pg/mL<br>*M3 (THS):* 3.2 (1.1-7.4)pg/mL<br>*M6 (THS):* 2.7 (0.5-8.1) pg/mL
[33]	Adults and children: Smokers and Nonsmokers with possible SHS exposure (NS) n = 5792, age: 6 - >60 years, 2964 female<br>06-11 years (NS n = 769)<br>12-19 (S n = 66, NS n=824)<br>20-59 (S n= 703, NS n=2123)<br>>60 (S n= 192, NS n=1115)<br>NHANES<br>(USA)	Serum	Cotinine<br>*Cut-off value*	*SHS:* <10 ng/mL
		Urine	NNAL<br>*GM(95% CI)*	*S (12-19 years):* 60.5 (46.2-79) pg/ mg cr<br>*S (20-59 years):* 209 (171-256) pg/ mg cr<br>*S (>60 years):* 365 (328-405) pg/ mg cr
				NS (6-11 years): 2.43 (1.96-3.02) pg/mg cr<br>*NS (12-19 years):* 1.38 (1.21-1.57) pg/mg cr<br>*NS (20-59 years):* 1.09 (1.00-1.19) pg/ mg cr<br>*NS (>60 years):* NA
[34]	Staff working in smoking hospitality venues: n= 62, 44 female<br>(Korea)	Urine	Cotinine<br>*GM (SD)*	*SHS:* 1.8 (2.8) ng/mg cr
			NNAL<br>*GM (SD)*	*SHS:* 7.3 (2.5) pg/mg cr
[35]	Adults: Smokers n = 69, SHS exposed n = 123, mean age: 56.7 years, men<br>(Japan)	Hair	Cotinine<br>*Mean (SD)*	*S*: 1.9 (2.1) ng/mg
				SHS: 0.2 (0.6) ng/mg
			Nicotine<br>*Mean (SD)*	*S*: 26.6 (24.7) ng/mg
				SHS: 3.6 (8.4) ng/mg
[36]	Adults: n = 10, mean age: 24.6 years, 20% female<br>Before exposure (BE) and after exposure (AE) during the work shift in waterpipe smoking bars<br>(USA)	Saliva	Cotinine<br>*Mean*	*BE (SHS):* 23.8 ng/mL<br>*AE(SHS):* 27.9 ng/mL
		Breath	CO<br>*Mean (SD)*	*BE (SHS):* 8.3 (6.9) ppm<br>*AE (SHS):* 49.4 (32.7) ppm
[37]	Mothers (A): Smokers n = 41, Nonsmokers n = 76<br>Daily S: Daily smokers n = 12<br>Oc. S: Ocassional smokers n = 11<br>Former S: Former smokers n = 18<br>NE: Not exposed - Nonsmoker n = 76<br><br>Children (C): n = 120, age: 6-11 years<br>Low SHS: less than daily exposure to SHS n = 6<br>High SHS: Daily exposure to SHS n = 6	Urine	Cotinine<br>*GM (95%CI)*	*A (Daily S)*: 489.15 (127.78-1872.44) µg/L<br>*A (Oc. S)*: 34.50 (6.35-187.55) µg/L
				A (Former S): 2.58 (1.51-4-39) µg/L<br>*A (NE)*: 1.41 (1.23-1.61) µg/L<br>*C (High SHS):* 10.77 (3.27-35.45) µg/L<br>*C (Low SHS):* 2.44 (1.26-4.71) µg/L<br>*C (NE)*: 1.39 (1.25-1.55) µg/L
			Cadmium<br>*GM (95%CI)*	*A (Daily S)*: 0.36 (0.30-0.44) µg/L<br>*A (Oc. S)*: 0.22 (0.15-0.33) µg/L

	NE: Not exposed n = 108<br><br>COPHES/DEMOCOPHES Projects<br>(Czech Republic)			*A (Former S):* 0.21 (0.16-0.28) µg/L<br>*A (NE)*: 0.21 (0.18-0.25) µg/L<br>*C (High SHS):* 0.180 (0.110-0.294) µg/L<br>*C (Low SHS):* 0.061 (0.028-0.131) µg/L<br>*C (NE)*: 0.110 (0.097-0.124) µg/L
[38]	Adults: n = 1398, age: 19-65 years, male and female<br>KNHANES<br>(Korea)	Urine	Cotinine<br>*Cut-off value*	*SHS:* < 550 µg/L
		Whole blood	Cadmium<br>*Age adjusted blood cadmium level*	*SHS*: 1.07 µg/L
[39]	Adolescents: n = 338, mean age: 12.9 years, 53% female<br>AdoQuest II longitudinal cohort<br>(Canada)	Saliva	Cotinine<br>*Mean (SD)*	*SHS*: 0.48 (1.21) ng/mL
		Hair	Nicotine<br>*Mean (SD)*	*SHS*: 0.38 (1.40) ng/mg
[40]	Children: n = 25, mean age: 5.4 years, male and female<br>Children with a potentially SHS-related illness with smoker parents<br>(USA)	Saliva	Cotinine<br>*Median (IQR)*	*SHS*: 5.3 (2.3-9.1) ng/mL
		Hand (wipes)	Nicotine<br>*Median (IQR)*	*SHS*: 91.6 (57.2-121.6) ng/wipe
[41]	Adults: Smokers n = 90/105, SHS exposed n = 91/103, S median age: 22 years, SHS meadian age: 27 years, male and female<br>Daily S: Daily smokers n= 17/20<br>Oc. S: Ocassional smokers n = 73/85<br>Before exposure (BE) and after exposure (AE) to waterpipe smoke in a waterpipe social event<br>(USA)	Saliva	Cotinine<br>*Cut-off value*	*SHS:* ≤ 10 ng/mL
		Urine	HPMA<br>*Median (IQR)*	*BE (Daily S):* 1443 (1131-3639) pmol/mg cr<br>*AE (Daily S)*: 3686 (2502-4046) pmol/mg cr<br>*BE (Oc. S):* 1660 (940-2890) pmol/mg cr<br>*AE (Oc. S)*: 2039 (1203-4898) pmol/mg cr
				BE: 1770 (1001-2787) pmol/mg cr<br>*AE (SHS):* 2498 (1580-3964) pmol/mg cr
[42]	Adolescents: Smokers n = 55, SHS exposed n = 410, age: 13-19 years, male and female<br>(USA)	Urine	Cotinine<br>*Median (IQR)*	*S:* > 30 ng/ mL
				Low SHS: 0.05-0.25 ng/mL<br>*High SHS:* 0.25-30 ng/ mL
			NNAL<br>*Median (IQR)*	*S*: 80.9 (42.6-151.3) pg/mL
				SHS: 1.2 (0.5-3.2) pg/mL
[43]	Adults and children: Smokers n = 867, Nonsmokers with possible SHS exposure (NS) n = 3825, age: 6->60 years, 53.4% female<br>NHANES<br>(USA)	Serum	Cotinine<br>*Cut-off range*	*SHS:* 0.05-10 ng/mL
		Urine	HPMM<br>*Median (IQR)*	*S*: 1.63 (0.680-3.29) mg/g cr
				NS: 0.313 (0.231-0.451) mg/g cr
[44]	Adults: n = 52-73, age: 24-40 years, male and female<br>Waterpipe Tobacco employees	Urine	Cotinine<br>*Median (IQR)*	*SHS*: 1.1 (0.2-40.9) µg/g cr

	(Turkey, Russia, Egipt)		NNAL<br>*Median (IQR)*	*SHS*: 1.48 (0.98-3.97) pg/mg cr
			1-OHPG<br>(1-hydroxy-pyrene glucuronide)<br>*Median (IQR)*	*SHS*: 0.54 (0.25-0.97) pmol/mg cr
		Saliva	Cotinine<br>*Median (IQR)*	*SHS*: 5.5 (2.0-15.0) ng/mL
		Hair	Nicotine<br>*Median (IQR)*	*SHS*: 0.95 (0.36-5.02) ng/mg
		Breath	CO<br>*Median (IQR)*	*SHS*: 1.67 (1.33-2.33) ppm

Abbreviations:

A: Adults; C: Children; NE: Nonsmokers not exposed to tobacco smoke; NS: Nonsmokers whose tobacco smoke exposure was not specified in the study; S: Smokers; SHS: Nonsmokers exposed to SHS; THS: Nonsmokers exposed to THS; AE: After exposure; Aft.: After smoking ban; BE: Before exposure; Bef.: Before smoking ban; BL: Baseline; Rest.: Restaurant; CI: Confidence interval; GM: Geometric mean; GSD: Geometric standard deviation; IQR: Interquartile range; LOD: Limit of detection; SD: Standard deviation; cr: Creatinine.

References

1. Jedrychowski, W. A.; Perera, F. P.; Maugeri, U.; Spengler, J.; Mroz, E.; Flak, E.; Stigter, L.; Majewska, R.; Kaim, I.; Sowa, A.; Jacek, R. Prohypertensive Effect of Gestational Personal Exposure to Fine Particulate Matter. Prospective Cohort Study in Non-smoking and Non-obese Pregnant Women. *Cardiovasc. Toxicol.* **2012**, *12*, 216–225, doi:10.1007/s12012-012-9157-z.
2. Protano, C.; Andreoli, R.; Manini, P.; Guidotti, M.; Vitali, M. A tobacco-related carcinogen: assessing the impact of smoking behaviours of cohabitants on benzene exposure in children. *Tob. Control* **2012**, *21*, 325–329, doi:10.1136/tc.2010.039255.
3. St.Helen, G.; Thomas Bernert, J.; Hall, D. B.; Sosnoff, C. S.; Xia, Y.; Balmes, J. R.; Vena, J. E.; Wang, J.-S.; Holland, N. T.; Naeher, L. P. Exposure to Secondhand Smoke Outside of a Bar and a Restaurant and Tobacco Exposure Biomarkers in Nonsmokers. *Environ. Health Perspect.* **2012**, *120*, 1010–1016, doi:10.1289/ehp.1104413.
4. Tzatzarakis, M. N.; Vardavas, C. I.; Terzi, I.; Kavalakis, M.; Kokkinakis, M.; Liesivuori, J.; Tsatsakis, A. M. Hair nicotine/cotinine concentrations as a method of monitoring exposure to tobacco smoke among infants and adults. *Hum. Exp. Toxicol.* **2012**, *31*, 258–265, doi:10.1177/0960327111422401.
5. Wilson, T.; Shamo, F.; Boynton, K.; Kiley, J. The impact of Michigan's Dr Ron Davis smoke-free air law on levels of cotinine, tobacco-specific lung carcinogen and severity of self-reported respiratory symptoms among non-smoking bar employees. *Tob. Control* **2012**, *21*, 593–595, doi:10.1136/tobaccocontrol-2011-050328.
6. Caman, O. K.; Erguder, B. I.; Ozcebe, H.; Bilir, N. Urinary Cotinine and Breath Carbon Monoxide Levels Among Bar and Restaurant Employees in Ankara. *Nicotine Tob. Res.* **2013**, *15*, 1446–1452, doi:10.1093/ntr/nts345.
7. Ho, G.; Tang, H.; Robbins, J. A.; Tong, E. K. Biomarkers of Tobacco Smoke Exposure and Asthma Severity in Adults. *Am. J. Prev. Med.* **2013**, *45*, 703–709, doi:10.1016/j.amepre.2013.09.002.
8. Joseph, A.; Spector, L.; Wickham, K.; Janis, G.; Winickoff, J.; Lindgren, B.; Murphy, S. Biomarker evidence of tobacco smoke exposure in children participating in lead screening. *Am. J. Public Health* **2013**, *103*, e54–e59, doi:10.2105/AJPH.2013.301315.
9. Lam, E.; Kvaavik, E.; Hamer, M.; Batty, G. D. Association of secondhand smoke exposure with mental health in men and women: Cross-sectional and prospective analyses using the UK Health and Lifestyle Survey. *Eur. Psychiatry* **2013**, *28*, 276–281, doi:10.1016/j.eurpsy.2012.04.001.

10. Leung, T. F.; Chan, I. H. S.; Liu, T. C.; Lam, C. W. K.; Wong, G. W. K. Relationship Between Passive Smoking Exposure and Urinary Heavy Metals and Lung Functions in Preschool Children. *Pediatr. Pulmonol.* **2013**, *48*, 1089–1097, doi:10.1002/ppul.22801.
11. Murphy, S. E.; Wickham, K. M.; Lindgren, B. R.; Spector, L. G.; Joseph, A. Cotinine and trans 3′-hydroxycotinine in dried blood spots as biomarkers of tobacco exposure and nicotine metabolism. *J. Expo. Sci. Environ. Epidemiol.* **2013**, *23*, 513–518, doi:10.1038/jes.2013.7.
12. Polanska, K.; Hanke, W.; Sobala, W.; Trzcinka-Ochocka, M.; Ligocka, D.; Brzeznicki, S.; Strugala-Stawik, H.; Magnus, P. Developmental Effects of Exposures to Environmental Factors: The Polish Mother and Child Cohort Study. *Biomed Res. Int.* **2013**, *ID 629716*, doi:10.1155/2013/629716.
13. Rajkumar, S.; Huynh, C. K.; Bauer, G. F.; Hoffmann, S.; Röösli, M. Impact of a smoking ban in hospitality venues on second hand smoke exposure: a comparison of exposure assessment methods. *BMC Public Health* **2013**, *13*, 536, doi:10.1186/1471-2458-13-536.
14. Schick, S. F.; Van Den Vossenberg, G.; Luo, A.; Whitlatch, A.; Jacob, P.; Balmes, J.; Shusterman, D. Thirty minute-exposure to aged cigarette smoke increases nasal congestion in nonsmokers. *J. Toxicol. Environ. Heal. - Part A Curr. Issues* **2013**, *76*, 601–613, doi:10.1080/15287394.2013.800811.
15. Szumska, M.; Tyrpień, K.; Kowalska, M.; Wielkoszyński, T.; Dobosz, C. Medicine students and exposure to environmental tobacco smoke. *Int. J. Occup. Med. Environ. Health* **2013**, *26*, 313–320, doi:10.2478/s13382-013-0100-8.
16. Vardavas, C. I.; Fthenou, E.; Patelarou, E.; Bagkeris, E.; Murphy, S.; Hecht, S. S.; Connolly, G. N.; Chatzi, L.; Kogevinas, M. Exposure to different sources of secondhand smoke during pregnancy and its effect on urinary cotinine and tobacco-specific nitrosamine (NNAL) concentrations. *Tob. Control* **2013**, *22*, 194–200, doi:10.1136/tobaccocontrol-2011-050144.
17. Matsumoto, A.; Matsumoto, A.; Ichiba, M.; Payton, N. M.; Oishi, H.; Hara, M. Simultaneous measurement of urinary total nicotine and cotinine as biomarkers of active and passive smoking among Japanese individuals. *Environ. Health Prev. Med.* **2013**, *18*, 244–250, doi:10.1007/s12199-012-0307-5.
18. Kim, S.; Apelberg, B. J.; Avila-Tang, E.; Hepp, L.; Yun, D.; Samet, J. M.; Breysse, P. N. Utility and Cutoff Value of Hair Nicotine as a Biomarker of Long-Term Tobacco Smoke Exposure, Compared to Salivary Cotinine. *Int. J. Environ. Res. Public Health* **2014**, *11*, 8368–8382, doi:10.3390/ijerph110808368.
19. Jones, I. A.; St Helen, G.; Meyers, M. J.; Dempsey, D. A.; Havel, C.; Jacob, P.; Northcross, A.; Hammond, S. K.; Benowitz, N. L. Biomarkers of secondhand smoke exposure in automobiles. *Tob. Control* **2014**, *23*, 51–57, doi:10.1136/tobaccocontrol-2012-050724.
20. Bentur, L.; Hellou, E.; Goldbart, A.; Pillar, G.; Monovich, E.; Salameh, M.; Scherb, I.; Bentur, Y. Laboratory and Clinical Acute Effects of Active and Passive Indoor Group Water-Pipe (Narghile) Smoking. *Chest* **2014**, *145*, 803–809, doi:10.1378/chest.13-0960.
21. Kim, H.-W.; Kam, S.; Lee, D.-H. Synergistic interaction between polycyclic aromatic hydrocarbons and environmental tobacco smoke on the risk of obesity in children and adolescents_ The U.S. National Health and Nutrition Examination Survey 2003–2008. **2014**, doi:10.1016/j.envres.2014.08.032.
22. Matt, G. E.; Quintana, P. J. E.; Fortmann, A. L.; Zakarian, J. M.; Galaviz, V. E.; Chatfield, D. a; Hoh, E.; Hovell, M. F.; Winston, C. Thirdhand smoke and exposure in California hotels: non-smoking rooms fail to protect non-smoking hotel guests from tobacco smoke exposure. *Tob. Control* **2014**, *23*, 264–272, doi:10.1136/tobaccocontrol-2012-050824.
23. Sánchez-Rodríguez, J. E.; Bartolomé, M.; Cañas, A. I.; Huetos, O.; Navarro, C.; Rodríguez, A. C.; Arribas, M.; Esteban, M.; López, A.; Castaño, A. Anti-smoking legislation and its effects on urinary cotinine and cadmium levels. *Environ. Res.* **2015**, *136*, 227–233, doi:10.1016/j.envres.2014.09.033.
24. Jain, R. B. Serum cotinine and urinary 4-(methylnitrosamino)-1-(3-pyridyl)-1-butanonol levels among non-Hispanic Asian American smokers and nonsmokers as compared to other race/ethnicities: Data from NHANES 2011-2012. *Chemosphere* **2015**, *120*, 584–591, doi:10.1016/j.chemosphere.2014.09.069.
25. Kim, J.; Kwon, H.-J.; Lee, K.; Lee, D.-H.; Paek, Y.; Kim, S.-S.; Hong, S.; Lim, W.; Heo, J.-H.; Kim, K. Air Quality, Biomarker Levels, and Health Effects on Staff in Korean Restaurants and Pubs Before and After a Smoking Ban. *Nicotine Tob. Res.* **2015**, *17*, 1337–1346, doi:10.1093/ntr/ntv012.
26. Pérez-Ortuño, R.; Martínez-Sánchez, J. M.; Fu, M.; Ballbè, M.; Quirós, N.; Fernández, E.; Pascual, J. A. Assessment of tobacco specific nitrosamines (TSNAs) in oral fluid as biomarkers of cancer risk: A population-based study. *Environ. Res.* **2016**, *151*, 635–641, doi:10.1016/j.envres.2016.08.036.
27. Northrup, T. F.; Khan, A. M.; Jacob, P.; Benowitz, N. L.; Hoh, E.; Hovell, M. F.; Matt, G. E.; Stotts, A. L. Thirdhand smoke contamination in hospital settings: Assessing exposure risk for vulnerable paediatric patients. *Tob. Control* **2016**, *25*, 619–623, doi:10.1136/tobaccocontrol-2015-052506.

28. Park, E. Y.; Yun, E. H.; Lim, M. K.; Lee, D.-H.; Yang, W.; Jeong, B. Y.; Hwang, S.-H. Consequences of incomplete smoke-free legislation in the Republic of Korea: Results from environmental and biochemical monitoring: Community based study. *Cancer Res. Treat.* **2016**, *48*, 376–383, doi:10.4143/crt.2014.269.
29. Okoli, C. A Comparison of Survey Measures and Biomarkers of Secondhand Tobacco Smoke Exposure among Nonsmokers. *Public Health Nurs.* **2016**, *33*, 82–89, doi:10.1111/phn.12207.
30. Pérez-Ortuño, R.; Martínez-Sánchez, J. M.; Fu, M.; Fernández, E.; Pascual, J. A. Evaluation of tobacco specific nitrosamines exposure by quantification of 4-(methylnitrosamino)-1-(3-pyridyl)-1-butanone (NNK) in human hair of non-smokers. *Sci. Rep.* **2016**, *6*, 1–8, doi:10.1038/srep25043.
31. Moore, B. F.; Clark, M. L.; Bachand, A.; Reynolds, S. J.; Nelson, T. L.; Peel, J. L. Interactions Between Diet and Exposure to Secondhand Smoke on Metabolic Syndrome Among Children: NHANES 2007-2010. *J. Clin. Endocrinol. Metab.* **2016**, *101*, 52–58, doi:10.1210/jc.2015-2477.
32. Matt, G. E.; Quintana, P. J. E.; Zakarian, J. M.; Hoh, E.; Hovell, M. F.; Mahabee-Gittens, M.; Watanabe, K.; Datuin, K.; Vue, C.; Chatfield, D. A. When smokers quit: exposure to nicotine and carcinogens persists from thirdhand smoke pollution. *Tob. Control* **2017**, *26*, 548–556, doi:10.1136/tobaccocontrol-2016-053119.
33. Wei, B.; Blount, B. C.; Xia, B.; Wang, L. Assessing exposure to tobacco-specific carcinogen NNK using its urinary metabolite NNAL measured in US population: 2011-2012. *J. Expo. Sci. Environ. Epidemiol.* **2016**, *26*, 249–256, doi:10.1038/jes.2014.88.
34. Kim, J.; Lee, K.; Kwon, H.-J.; Lee, D. H.; Kim, K. Association between Secondhand Smoke in Hospitality Venues and Urinary 4-(methylnitrosamino)-1-(3-pyridyl)-1-butanol Concentrations in Non-Smoking Staff. *Int. J. Environ. Res. Public Health* **2016**, *13*, doi:10.3390/ijerph13111101.
35. Tsuji, M.; Kanda, H.; Hayakawa, T.; Mori, Y.; Ito, T.; Hidaka, T.; Kakamu, T.; Kumagai, T.; Osaki, Y.; Kawazoe, M.; Sato, S.; Fukushima, T. Nicotine cut-off value in human hair as a tool to distinguish active from passive smokers: A cross-sectional study in Japanese men. *Cancer Biomarkers* **2017**, *20*, 41–48, doi:10.3233/CBM-170004.
36. Zhou, S.; Behrooz, L.; Weitzman, M.; Pan, G.; Vilcassim, R.; Mirowsky, J. E.; Breysee, P.; Rule, A.; Gordon, T. Secondhand hookah smoke: An occupational hazard for hookah bar employees. *Tob. Control* **2017**, *26*, 40–45, doi:10.1136/tobaccocontrol-2015-052505.
37. Forysová, K.; Pinkr-Grafnetterová, A.; Malý, M.; Krsková, A.; Mráz, J.; Kašparová, L.; Čejchanová, M.; Sochorová, L.; Rödlová, S.; Černá, M. Urinary Cadmium and Cotinine Levels and Hair Mercury Levels in Czech Children and Their Mothers Within the Framework of the COPHES/DEMOCOPHES Projects. *Arch. Environ. Contam. Toxicol.* **2017**, *73*, 421–430, doi:10.1007/s00244-017-0412-y.
38. Lee, W.; Lee, S.; Roh, J.; Won, J. U.; Yoon, J. H. The Association between Involuntary Smoking Exposure with Urine Cotinine Level and Blood Cadmium Level in General Non-Smoking Populations. *J. Korean Med. Sci.* **2017**, *32*, 568–575, doi:10.3346/jkms.2017.32.4.568.
39. McGrath, J. J.; Racicot, S.; Okoli, C. T. C.; Hammond, S. K.; O'Loughlin, J. Airborne Nicotine, Secondhand Smoke, and Precursors to Adolescent Smoking. *Pediatrics* **2018**, *141*, S63–S74, doi:10.1542/peds.2017-1026J.
40. Mahabee-Gittens, E. M.; Merianos, A. L.; Matt, G. E. Preliminary evidence that high levels of nicotine on children's hands may contribute to overall tobacco smoke exposure. *Tob. Control* **2018**, *27*, 217–219, doi:10.1136/tobaccocontrol-2016-053602.
41. Kassem, N. O. F.; Kassem, N. O.; Liles, S.; Zarth, A. T.; Jackson, S. R.; Daffa, R. M.; Chatfield, D. A.; Carmella, S. G.; Hecht, S. S.; Hovell, M. F. Acrolein Exposure in Hookah Smokers and Non-Smokers Exposed to Hookah Tobacco Secondhand Smoke: Implications for Regulating Hookah Tobacco Products. *Nicotine Tob. Res.* **2018**, *20*, 492–501, doi:10.1093/ntr/ntx133.
42. Benowitz, N. L.; Nardone, N.; Jain, S.; Dempsey, D. A.; Addo, N.; St. Helen, G.; Jacob, P. Comparison of Urine 4-(Methylnitrosamino)-1-(3)Pyridyl-1-Butanol and Cotinine for Assessment of Active and Passive Smoke Exposure in Urban Adolescents. *Cancer Epidemiol. Biomarkers Prev.* **2018**, *27*, 254–261, doi:10.1158/1055-9965.EPI-17-0671.
43. Bagchi, P.; Geldner, N.; DeCastro, B. R.; De Jesús, V. R.; Park, S. K.; Blount, B. C. Crotonaldehyde exposure in U.S. tobacco smokers and nonsmokers: NHANES 2005–2006 and 2011–2012. *Environ. Res.* **2018**, *163*, 1–9, doi:10.1016/j.envres.2018.01.033.
44. Moon, K. A.; Rule, A. M.; Magid, H. S.; Ferguson, J. M.; Susan, J.; Sun, Z.; Torrey, C.; Abubaker, S.; Levshin, V.; Çarkoglu, A.; Radwan, G. N.; El-Rabbat, M.; Cohen, J. E.; Strickland, P.; Breysse, P. N.; Navas-Acien, A. Biomarkers of secondhand smoke exposure in waterpipe tobacco venue employees in Istanbul, Moscow, and Cairo. *Nicotine Tob. Res.* **2018**, *20*, 482–491, doi:10.1093/ntr/ntx125.
